# RASON promotes KRAS^G12C^-driven tumor progression and immune evasion in non-small cell lung cancer

**DOI:** 10.1186/s13046-025-03369-9

**Published:** 2025-03-25

**Authors:** Jianzhuang Wu, Kexin Xie, Yixuan Zhang, Weiyi Zhang, Rongjie Cheng, Yaliang Zhang, Yugui Xia, Tongyan Liu, Rong Yin, Yudong Qiu, Tao Xu, Rutian Li, Qi Sun, Chao Yan

**Affiliations:** 1 Department of Pancreatic and Metabolic Surgery, Nanjing Drum Tower Hospital, Affiliated Hospital of Medical School, Nanjing University, Nanjing, China; 2 State Key Laboratory of Pharmaceutical Biotechnology, School of Life Sciences, Nanjing University, Nanjing, China; 3 Institute of Artificial Intelligence Biomedicine, Nanjing University, Nanjing, China; 4 Cancer Stem Cell Laboratory, The Breast Cancer Now Toby Robins Research Centre, The Institute of Cancer Research, London, UK; 5 Department of Thoracic Surgery, Jiangsu Key Laboratory of Molecular and Translational Cancer Research, Jiangsu Cancer Hospital and Nanjing Medical University Affiliated Cancer Hospital and Jiangsu Institute of Cancer Research, Nanjing, China; 6 Collaborative Innovation Centre for Cancer Personalized Medicine, Nanjing Medical University, Nanjing, China; 7 Inflammation and Immune Mediated Diseases Laboratory of Anhui Province, Anhui Institute of Innovative Drugs, School of Pharmacy, Anhui Medical University, Hefei, China; 8 The Comprehensive Cancer Center, Nanjing Drum Tower Hospital, Affiliated Hospital of Medical School, Nanjing University, Nanjing, China; 9 Department of Pathology, Nanjing Drum Tower Hospital, The Affiliated Hospital of Nanjing University Medical School, Nanjing, China

**Keywords:** RASON, KRAS, Non-small cell lung cancer, Immune evasion, CD47

## Abstract

**Background:**

KRAS is the most frequently mutated oncogene in human cancers, with KRAS^G12C^ being a prevalent driver mutation in 12–13% non-small cell lung cancer (NSCLC) cases. Despite breakthroughs in KRAS^G12C^ inhibitors such as sotorasib (AMG-510) and adagrasib (MRTX-849), clinical resistance remains a challenging issue, highlighting the need for deeper understanding of the molecular mechanisms underlying KRAS^G12C^-driven oncogenic signaling in NSCLC. Previously, we identified RASON as a novel regulator of KRAS^G12D/V^ signaling in pancreatic cancer. Herein, we aim to explore the role of RASON in KRAS^G12C^-driven NSCLC and its therapeutic potential.

**Methods:**

Immunohistochemistry analysis of NSCLC patient cohorts was performed to demonstrate the correlation between RASON expression and NSCLC progression. Immunoblotting was performed to evaluate the effects of RASON on KRAS^G12C^ downstream signaling. In vitro and in vivo assays including cell proliferation, sphere formation, tumor implantation and genetic mouse models were performed to determine the oncogenic role of RASON. RNA-seq analysis was utilized to identify the key signaling pathway regulated by RASON. Immunofluorescence, immunoprecipitation, nuclear magnetic resonance and biochemistry assays were used to validate the interaction between KRAS^G12C^ and RASON. Phagocytosis assay and flow cytometry were conducted to explore the effects of RASON on the tumor immune microenvironment. Pharmacological inhibition in subcutaneous xenograft model was used to determine the therapeutical potential of RASON.

**Results:**

RASON is overexpressed in NSCLC with KRAS^G12C^ mutation and correlates with poor patient prognosis. Genetic knockout of RASON significantly reduced lung tumor burden in LSL-KRAS^G12D^; Trp53^R172H/+^ mice. In KRAS^G12C^-mutant lung cancer cell lines, RASON overexpression enhanced, while CRISPR-mediated knockout suppressed, both in vitro proliferation and in vivo tumor growth. Mechanistically, RASON directly binds KRAS^G12C^, stabilizes it in the GTP-bound hyperactive state and promotes downstream signaling. RASON knockout significantly reduced CD47 expression, enhancing macrophage-mediated phagocytosis and anti-tumor immunity. Therapeutically, antisense oligonucleotides targeting RASON not only exhibited tumor-suppressive effects, but also synergized with the KRAS^G12C^ inhibitor AMG-510 to significantly enhance anti-tumor efficacy.

**Conclusion:**

This study reveals RASON as a key oncogenic regulator of KRAS^G12C^ signaling, driving lung tumorigenesis and progression, and identifies RASON as a promising therapeutic target for KRAS^G12C^ mutant non-small cell lung cancer.

**Supplementary Information:**

The online version contains supplementary material available at 10.1186/s13046-025-03369-9.

## Introduction

Lung cancer remains the most common cancer type worldwide, accounting for approximately 1.6 million deaths annually [1, 2]. Non-small cell lung cancer (NSCLC) represents about 85% of all lung cancers, with lung adenocarcinoma (LUAD) and lung squamous cell carcinoma (LUSC) being the most prevalent histological subtypes. Among the various oncogenes implicated in NSCLC, mutations in the *KRAS* gene are detected in around 33.6% of cases [3–5].

The rat sarcoma (*RAS*) gene family, comprising *HRAS*,* KRAS* and *NRAS*, is one of the most frequently mutated oncogene families in human cancers. *KRAS* is the most commonly mutated isoform, accounting for about 85% of RAS-driven malignancies. Notably, *KRAS* mutations are detected in approximately 90% of pancreatic cancers, 50% of colorectal cancers, and 30% of lung cancers [6–8]. Within NSCLC, the KRAS^G12C^ mutation constitutes around 40% of all *KRAS* mutations [9–11]. KRAS proteins function as molecular switches, cycling between inactive (GDP-bound) and active (GTP-bound) states to regulate key signaling pathways [12]. Oncogenic KRAS mutations lock the protein in its active state, resulting in persistent activation of downstream signaling cascades, such as the mitogen-activated protein kinase (MAPK) and phosphatidylinositol 3-kinase (PI3K) pathways, which drive tumor growth and progression [13–16].

Targeting KRAS has historically been challenging due to its high affinity for GTP and the lack of a druggable binding pocket [17]. However, recent advances in our understanding of the chemical biology of KRAS^G12C^ signaling have led to the development of covalent inhibitors targeting the cysteine residue unique to the KRAS^G12C^ mutation [18–21]. Among these, sotorasib (AMG-510) [18] and adagrasib (MRTX-849) [19] have received FDA approval and shown clinical efficacy in KRAS^G12C^ mutant NSCLC. Despite these breakthroughs, intrinsic and acquired resistance remain significant barriers, limiting their long-term therapeutic success [22–30]. This underscores the need for a deeper understanding of the molecular mechanisms governing KRAS^G12C^ signaling.

Recently, we reported the discovery of RASON, a novel protein encoded by the long non-coding RNA *LINC00673*, as an oncogenic regulator of KRAS in pancreatic ductal adenocarcinoma (PDAC) [31]. RASON stabilizes KRAS^G12D^ and KRAS^G12V^ mutations in their GTP-bound active states, promoting downstream signaling and PDAC progression. In this study, we reveal that RASON also plays a critical role in KRAS^G12C^-driven NSCLC. RASON directly binds to and promotes the activation of KRAS^G12C^, leading to constitutive activation of oncogenic KRAS signaling. Importantly, pharmacological inhibition of RASON not only suppresses tumor growth but also synergistically enhances the efficacy of the KRAS^G12C^ inhibitor AMG-510 in preclinical models. These findings establish RASON as a promising therapeutic target both as monotherapy and synergistically with KRAS inhibitors to improve outcomes in KRAS^G12C^-mutant NSCLC.

## Methods

### Mice

All animal care and handling procedures were performed in accordance with the National Institutes of Health’s Guide for the Care and Use of Laboratory Animals, with ethical approval by the Ethical Committee of Nanjing University (IACUC-2209002).

Transgenic *Kras*^LSL−G12D^; *Trp53*^R172H/+^ (KP) and *Kras*^LSL−G12D^; *Trp53*^R172H/+^; *Rason*^mut/mut^ (KPR) mouse models were generated and obtained from GemPharmatech Co. Ltd. C57B6/J and BALB/c nude mice were obtained from Cyagen Biosciences, Inc.

### Lung tumor models in KP and KPR mice

For KRAS^G12D^ activation in mouse lungs, KP and KPR mice were first anaesthetized with 1.25% tribromoethanol (0.2 mL/10 g), followed by nasal inhalation of 1 × 10^10^ plaque-forming units of AAV-Cre diluted in 50 µL PBS. Mice in the KP or KPR group were randomly divided into two subgroups to monitor the survival time and tumor growth, respectively. For survival analysis, mice were monitored for 150 days without further treatment. To monitor tumor growth, mice were anesthetized, and tumor size was measured by micro-computed tomography (micro-CT) scanning. Two days after scanning, the mice were euthanized for histological confirmation of lung adenocarcinoma by hematoxylin and eosin (H&E) staining. Excised tumors were analyzed for RASON expression and macrophage infiltration using immunoblotting, immunofluorescence staining, and immunohistochemistry.

### Subcutaneous xenograft models

Six-week-old male BALB/c nude mice were used to establish the subcutaneous tumor models. Tumor cells were collected, resuspended in cold PBS, and mixed with Matrigel on ice. 1 × 10^6^ cells were injected subcutaneously into the dorsal region of each mouse using insulin needles (29G, BD). Tumor volume was calculated as V = 0.52×length×width^2^. For treatment experiments, mice were randomly divided into four groups at two weeks after injection: control, AMG-510 monotherapy (P.O., 30 mg/kg, QD), RASON-ASO monotherapy (I.P., 50 nmol, QD), and combination therapy with AMG-510 and RASON-ASO. Mice were sacrificed on the thirtieth day to evaluate the treatment effects.

### Lung colonization models

One million control or RASON-KO LLC cells were resuspended in 100 µL PBS and intravenously injected into C57B6/J mice using insulin needles (29G, BD). One month after the injection, the mice were sacrificed and the primary tumors and lungs were excised to assess the tumor areas using H&E staining.

### Cells

Human lung cancer cell lines H23, H358, H1792, H2122, and the mouse lung cancer cell line LLC were obtained from ATCC. KRAS^G12C^-transformed Ras-less mouse embryonic fibroblast cells (MEF^G12C^) were obtained from the NIH RAS Initiative and cultured as indicated (https://www.cancer.gov/research/key-initiatives/ras/tools-resources). H23, H358, H1792, and H2122 cells were maintained in RPMI-1640 medium supplemented with 10% fetal bovine serum (FBS) and 1% penicillin-streptomycin (PS). LLC and RAS-less MEF were maintained in DMEM supplemented with 10% FBS and 1% PS. All the cell lines were incubated in a humidified atmosphere containing 5% CO_2_ at 37 °C. Cells were regularly tested for mycoplasma contamination.

### Human samples

Samples from three patient cohorts were used for RASON expression and correlation analyses between RASON and CD47 levels. The first cohort was a commercial tissue microarray containing 80 pairs of lung adenocarcinomas. Normal adjacent tissue samples were produced from 160 paraffin-embedded samples by Aifang Biotechnology (Changsha, Hunan, China). The second cohort containing 44 KRAS-mutant and 91 KRAS-WT NSCLC patients was collected from Nanjing Drum Tower Hospital with the patients’ written consent under a protocol approved by the Institutional Review Board of the institution and confirmed by a pathologist before use.The third cohort was an in-house generated tissue microarray containing nine KRAS^mut^ human NSCLC tissue samples. These samples were obtained from the Jiangsu Biobank of Clinical Resources (Jiangsu Cancer Hospital, Nanjing, China) with the patients’ written consent under a protocol approved by the Institutional Review Board of the institution and confirmed by a pathologist before use.

### Micro-CT scanning

Micro-CT analysis was performed to assess lung tumor growth in KP and KPR mice, and 3-D pulmonary images were reconstructed. Briefly, micro-CT scans were performed using a Hiscan micro-CT analyzer. All scans were performed with an 80-kV tube voltage and a 100-A tube current, capturing images at a 25-µm resolution. A 0.5° rotation step was employed over a 360° angular range, with a 50-ms exposure per step. Image reconstruction was performed using Hiscan Reconstruct software (version 3.0, Suzhou Hiscan Information Technology), and subsequent analysis of the original three-dimensional (3D) images of the lung tumors was performed using Hiscan Analyzer software (version 3.0, Suzhou Hiscan Information Technology).

### Histopathology

For histopathological examination of tumor xenografts or surgical specimen tissues, whole lungs were fixed overnight in 4% paraformaldehyde and embedded in paraffin. H&E staining was performed according to the standard protocols. Briefly, paraffin-embedded tissue sections were deparaffinized, rehydrated, and immersed in hematoxylin solution for 1–2 min at room temperature. After rinsing with distilled water, the slides were counterstained with eosin. Digitally scanned images of the H&E-stained slides were captured using an Olympus VS120 system or an Olympus Bx53 microscope at 20x magnification for analysis. Tumor regions were outlined, and the percentage of the tumor area relative to the total lung area was calculated for each mouse.

### Immunofluorescence staining

Cells were fixed for 10 min in 4% PFA and permeabilized with PBST (PBS with 0.5% Triton X-100). The cells were then blocked with 5% bovine serum albumin with 0.25% Triton X-100 in PBS prior to incubation with RASON (GenScript) and KRAS primary antibodies (diluted 1:200) overnight at 4 °C, followed by three washes with PBST. Subsequently, the cells were incubated with Alexa Fluor 488 or 568–conjugated secondary antibodies (diluted 1:800 in blocking solution) for 1 h at room temperature. Nuclei were counterstained with 4′,6-diamidino-2-phenylindole (DAPI). Images were captured using a Leica SP8 confocal microscope and analyzed using the LAS X software and ImageJ.

### FACS analysis

Tumors from the lung colonization model were minced into sub-millimeter-sized pieces and placed in 0.8 mL RPMI-1640 with Liberase TL (0.2 mg/mL) and DNase I (20 µg/mL) for 1 h. The specimens were then passed through a 70 mm mesh and centrifuged at 350 g for 5 min. Cell pellets were resuspended, and cell labeling was performed by incubating 1 × 10^6^ cells with 0.5 µg fluorescently conjugated antibodies against CD3, CD4, CD8a, CD45, CD11b, F4/80, CD206, and CD86, following the intracellular staining protocol. Cells were fixed in 4% paraformaldehyde, washed, resuspended in PBS, and analyzed using flow cytometry (Attune NxT, Thermo Fisher).

### Immunohistochemistry

Immunohistochemistry was performed according to standard protocols. Before staining, 5-µm paraffinized lung tumor sections were baked at 60 °C for 1 h, deparaffinized in xylene, and rehydrated through a graded ethanol series. Slides were incubated in H_2_O_2_ for 5 min and subjected to antigen retrieval using a 10 mM citrate solution in a heated pressure cooker for 10 min. Tissue sections were blocked in an animal-free blocker and incubated overnight at 4 °C with monoclonal antibodies against RASON, CD47, CD11b, p-ERK, and p-AKT. The primary antibodies were detected using HRP-conjugated rabbit secondary antibodies and visualized using a DAB Impact kit. After staining, the slides were dehydrated using a graded ethanol series, cleared in xylene, and mounted. Images were captured using an Olympus VS120 system or Olympus Bx53 microscope at 20× magnification and analyzed using associated software (OlyVIA, version 4.1).

### RNA interference (RNAi)

Cells were transfected with 50 nM RASON siRNA, firefly luciferase siRNA (negative control), or scrambled siRNA (negative control). Cells were seeded in 6-well plates and transfected using Lipofectamine 3000 reagent according to the manufacturer’s protocol. Total protein was extracted at 24–48 h after transfection. The sequences of synthetic siRNAs and reagents used in this study are listed in Table S1 and Table S2.

### Generation of stable cell lines

#### CRISPR/CAS9-mediated knockout

Stable knockout cell lines were generated by lentiviral transduction. The sgRNA sequences targeting RASON (homo, TTGGATGGAAAGTGGGGAAT; mus, CGCGGATTGTGTGTTTGCGT, and GGGTTGGGGTTCCCCTAACG) were designed using the online tool http://crispr.mit.edu/. CRISPR-lentivirus was produced according to the manufacturer’s instructions. Next, 10^5^ cells per well were seeded into 24-well plates and incubated in a humidified atmosphere with 5% CO_2_ at 37 °C. After 12 h, the medium was replaced with 0.5 mL Opti-MEM culture medium (without FBS and PS) containing 5 × 10^5^ TU lentivirus (MOI = 50). After 24 h of transfection, the medium was replaced with normal DMEM or RPMI-1640 supplemented with 10% FBS and 1% PS. Transfected cells were counted, diluted, and seeded in 96-well plates to generate monoclonal cells. Genomic DNA was extracted from the monoclonal cell populations, and the genotypes of the mono-clones were verified and selected via sequencing.

### shRNA-mediated knockdown

To generate stable RASON-knockdown cell lines, 10^5^ cells per well were seeded in 24-well plates and cultured in 5% CO_2_ at 37 °C. After 12 h, the medium was replaced with 0.5 mL Opti-MEM culture medium containing 5 × 10^5^ TU lentivirus (MOI = 50). After 24 h of transfection, the medium was replaced with normal DMEM or RPMI-1640 medium containing 10% FBS and 1% PS. Two days after cell passaging, the culture medium was replaced with 2 µg/mL puromycin or 1 µg/mL G418 (in DMEM or RPMI-1640) to select transfected cells. Stable multiclonal cell lines were maintained in a complete medium containing 2 µg/mL puromycin or 1 µg/mL G418.

### In vitro phagocytosis assay

Peripheral blood mononuclear cells (PBMCs) were isolated from healthy donors using density gradient centrifugation with Ficoll-Hypaque. Isolated PBMCs were cultured in complete RPMI-1640 medium (supplemented with 2 mmol/L glutamine, and 10% FBS) and stimulated with granulocyte-macrophage colony-stimulating factor (GM-CSF) at 25 ng/mL for 7 days to generate macrophages. The phagocytosis assay was conducted as previously described. Briefly, macrophages were seeded at a density of 5 × 10^4^ cells/well in a 24-well tissue culture plate in complete DMEM supplemented with GM-CSF overnight before the experiment. H358 cells were labeled with 2.5 µM carboxyfluorescein diacetate succinimidyl ester (CFSE) at 37 °C for 10 min. Macrophages were pre-incubated in serum-free medium for 2 h before co-culturing with 2 × 10^5^ CFSE-labeled H358 cells. After 2 h of co-culture at 37 °C, the cells were harvested and stained with 0.2 µg PE-labelled anti-CD14 antibody. Flow cytometry (Attune NxT, Thermo Fisher) was performed to detect CFSE + CD14 + cells.

### Protein extraction and Immunoblotting

Cells were lysed in RIPA buffer (0.5% NP-40, 0.1% sodium deoxycholate, 150 mM NaCl, and 50 mM Tris-HCl, pH 7.5) supplemented with a protease and phosphatase inhibitor cocktail. The protein concentration was determined using the Pierce BCA Protein Assay Kit. Equal amounts of protein were resolved using 12.5% or 15% SDS-PAGE and then transferred to a PVDF membrane (0.45 μm). The membrane was blocked with 5% nonfat milk in 0.1% TBST and incubated overnight with the corresponding primary antibodies at 4 °C. The membrane was washed six times with TBST. After 1 h incubation with secondary antibodies at room temperature, protein detection was performed using a chemiluminescent reagent. Band intensities were quantified using the ImageJ software. The primary antibodies used for western blotting were KRAS (1:1000), AKT (1:1000), p-AKT (1:1000), ERK (1:1000), p-ERK (1:1000), and β-actin (1:5000). The secondary antibodies used were Goat Anti-Rabbit IgG (H + L) HRP and HRP-conjugated Recombinant Rabbit Anti-Mouse IgG Kappa Light Chain.

### Protein expression and purification

KRAS-G12C and SOS1 coding sequence was cloned into the pET-30a (+) vector. RASON coding sequence was cloned into the pET28a vector. The coding sequence of GAP-related domain (GRD) of NF1 protein was cloned into the pET-24d vector.

For prokaryotic expression, proteins were expressed and purified from *E.coli* BL21 (DE3) cell extracts by affinity purification followed by size exclusion chromatography. In brief, protein expression was induced by 1 mM isopropyl-β-D-1-thiogalactopyranoside at 37 °C for 4 h. Cells were collected by centrifugation and resuspended in lysis buffer (50 mM Tris-HCl, pH 7.4, 500 mM NaCl, 1 mM TCEP). The suspension was lysed by sonication in an ice-water bath and centrifuged at 13,000 g for 30 min at 4 °C. The supernatant was incubated with Ni-NTA for 20 min, washed and eluted with 200 mM imidazole. Proteins were further purified by Superdex 75 10/300 GL gel-filtration column (GE Healthcare) and stored in 50 mM HEPES, pH 7.4, 150 mM NaCl, 1 mM TCEP, 1 mM MgCl_2_ at -80℃.

For RASON purification, the inclusion bodies were washed twice in lysis buffer, dissolved in 8 M urea and underwent affinity purification by Ni NTA Beads (Smart-Lifesciences). The elution containing fusion protein was allowed to refold by dialysis. RASON protein was obtained after cleaving off the recombinant tag by TEV protease and was further purified using Superdex 75 10/300 GL gel-filtration column (GE Healthcare).

For NMR experiments, 15 N-labeled KRAS-G12C protein was produced in *E. coli* BL21 (DE3) which was cultured in M9 medium (^15^NH_4_Cl) with 5 g/L glucose, 2 mM MgSO_4_, and 0.1 mM CaCl_2_, and purified as mentioned above.

### NMR experiment

NMR experiments were carried out at 298 K on a Bruker AVANCE NEO 800 MHz spectrometer. The 2D ^1^H-^15^N HSQC spectra of 15 N-labeled KRAS-G12C with RASON protein at indicated molar ratios were obtained to detect the interaction. All NMR spectra were processed and plotted using topspin 4.1.1.

### GTP hydrolysis assay

GTP hydrolysis rates of KRAS-G12C was measured by detecting the production of inorganic phosphates as previously described [32]. Briefly, KRAS-G12C protein was loaded with 10 mM GTP (Sigma, MO, USA, 11140957001) for 2 h at 4 °C. Each reaction contains 2 µM GTP-loaded KRAS protein in reaction buffer (10 mM Tris, pH 7.6, 150 mM NaCl, 2 mM MgCl_2_, 0.05% Tween-20). For intrinsic hydrolysis, reaction was started by adding SOS1 (final concentration 1 µM) and RASON (final concentrations 0.0 µM, 0.1 µM, 0.3 µM, 1.0 µM, 3.0 µM, 10.0 µM). For extrinsic hydrolysis, NF1 (RAS-GAP) was added additionally (final concentration 1 µM). The reaction mixture was allowed to incubated at 37 °C for indicated time and 100 µM EDTA was added to stop the reaction.

The optical absorption was measured using a Spectramax M4 plate reader at 630 nm (Molecular Device, San Jose, USA) after adding 20 µL color reagent (1 mg/mL Malachite Green, 3 mg/mL Ammonium molybdate tetrahydrate, 0.12% Tween-20 in 1 N sulfuric acid) to 10 µL reaction mixture in each well of 384-well plate. Samples were measured in triplicates and the hydrolysis curve was fitted using one phase association model in GraphPad Prism 8 (San Diego, CA).

### Nucleotide exchange assay

Nucleotide exchange activity of KRAS-G12C was carried out according to a published protocol [33]. Briefly, KRAS proteins (10 µM) were loaded with 200 µM mant-GDP (Sigma, MO, USA, 69244) in the presence of 2.5 mM EDTA (Sigma, E5134) in reaction buffer (20 mM HEPES, pH 7.5, 150 mM NaCl, 1 mM DTT and 1 mM MgCl_2_). After incubation for 1 h at room temperature in dark, loading reaction was terminated by adding 10 mM MgCl_2_. The proteins were desalted using NAP-5 columns (GE, Chicago, USA, 17085302). 10 µL of each desalted protein was added to low-volume black bottom 384-well plates (Corning, NY, USA, 4514). To initiate the nucleotide exchange reaction, GMPPNP (1 mM final) (sigma, G0635), EDTA (5 mM final) and 5 µL RASON protein (0.0 µM, 0.3 µM, 1.0 µM, 3.0 µM, 10.0 µM final) with (for extrinsic exchange) or without (for intrinsic exchange) SOS (1 µM final) was added and fluorescence was monitored on a Spectramax M4 plate reader (Molecular Device, San Jose, USA) (355 nm excitation, 448 nm emission) for 5 h at 90-s intervals. Exchange curve was fitted using one phase decay model in GraphPad Prism 8 (San Diego, CA).

### Cell proliferation assay

Cell proliferation was assessed using the CCK8 assay (Yeasen, #40203ES88). Two thousand cells per well were seeded in 96-well plates and cultured in complete medium for the indicated time. Each experiment was performed in triplicate. Cell numbers were determined by measuring absorbance at 450 nm, following the manufacturer’s protocol.

### 2D colony formation assay

Three hundred cells per well were seeded in 6-well plates and incubated in complete medium for 2 weeks. The colonies were then fixed with 4% paraformaldehyde for 20 min, stained with 0.1% crystal violet for 20 min, and observed under a microscope.

### Immunoprecipitation

Immunoprecipitation was conducted using an Immunoprecipitation Kit, according to the manufacturer’s protocol. Briefly, Protein A&G magnetic beads were incubated with 5 µg KRAS primary antibodies overnight at 4 °C. The beads were washed once with wash buffer to remove unbound antibodies. Cells were lysed in lysis buffer. The soluble fractions from the cell lysates were added and incubated with the beads for 2 h at room temperature. The beads were washed three times with IP Wash Buffer and eluted with SDS Sample Buffer. Immunoprecipitants were analyzed by immunoblotting.

### RAS activity pulldown assay

GTP-bound KRAS (active KRAS) was measured using the C-Raf RAS-binding-domain (RBD) pull-down and detection kit (Cytoskeleton) following the manufacturer’s instructions. In brief, cell lysates were mixed with GST-Raf-RBD and glutathione resin and incubated at 4℃ for 30 min. The bound proteins were then eluted and analyzed by immunoblotting, with total RAS sample added as a control.

### RNA-seq analysis

Total RNA was extracted from cultured cells using TRIzol Reagent according to the manufacturer’s instructions. RNA purification, reverse transcription, library construction, and sequencing were performed by Shanghai Majorbio Bio-Pharm Biotechnology Co., Ltd. (Shanghai, China). The transcriptome library for RNA-seq was generated using 1 µg of total RNA with the Illumina^®^ Stranded mRNA Prep Ligation kit from Illumina (San Diego, CA, USA). After quality control of the raw reads, the clean reads were mapped using HISAT2 software and further assembled using String Tie. Differential expression analysis between H358 and H358 RASON knockout cells was performed using the R (v.4.3.0) package, DESeq2 (v.1.24.0). Cellular pathway analysis was performed using GSEA (v.4.3.2). The data were further analyzed using the online platform of Majorbio Cloud Platform (www.majorbio.com).

### Statistical analysis

All statistical analyses were performed using R or GraphPad Prism 8 (San Diego, CA). Data are presented as the mean ± S.E.M. or mean ± S.D. Differences were considered statistically significant at *p* < 0.05. Normality and equal variances among group samples were assessed using the Shapiro-Wilk test and the Brown–Forsythe test, respectively. When normality and equal variance were confirmed, *one-way ANOVA* (followed by Bonferroni’s multiple comparisons test), *two-way ANOVA*, or *Student’s t-test* were used.

### Data Availability

RNA-seq data from this study were deposited in the NCBI Sequence Read Archive (SRA, PRJNA1199328) and will be publicly available as of the date of publication.

## Results

### RASON is essential for KRAS-driven lung tumorigenesis in mice

**Fig. 1:**
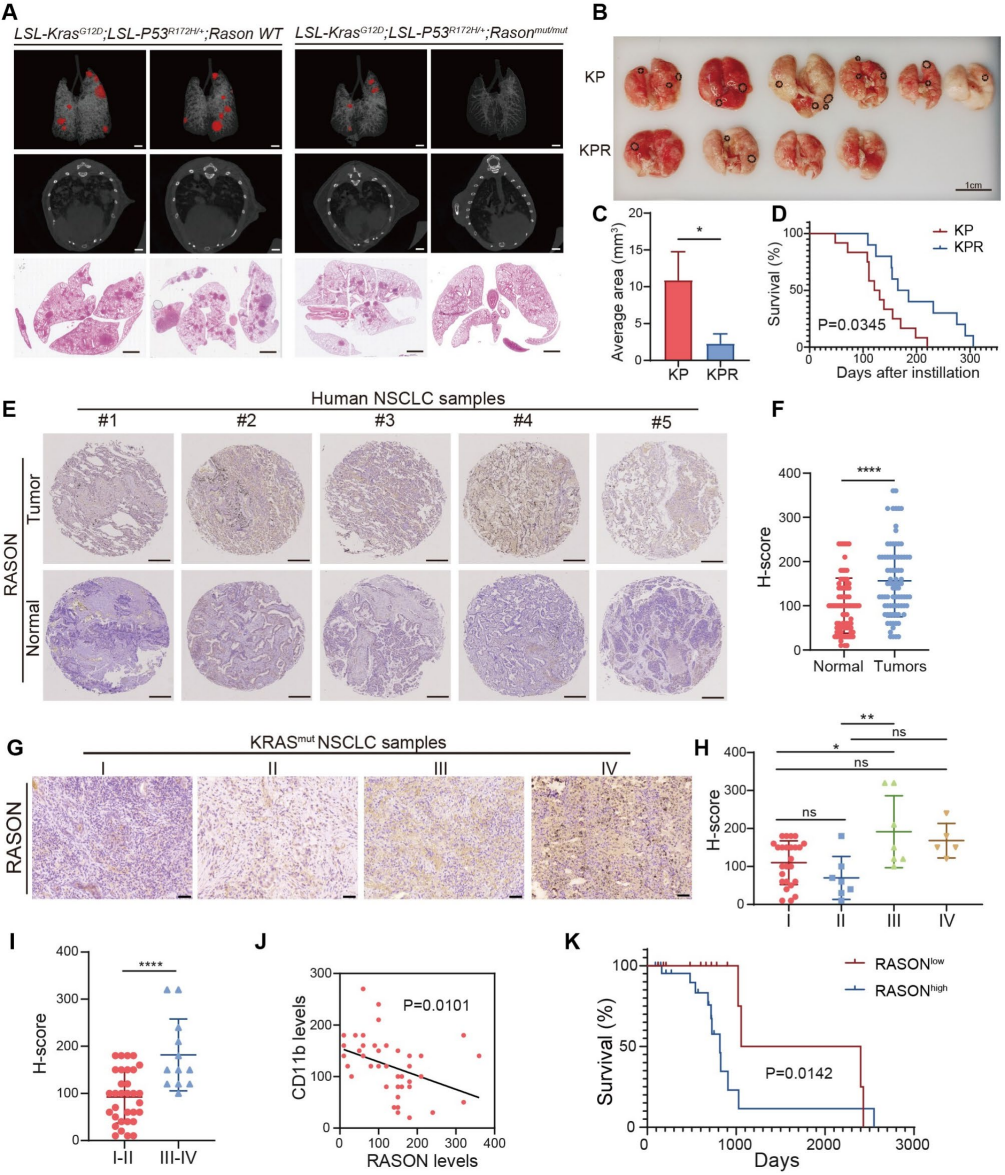
RASON is essential for KRAS-driven lung tumorigenesis in mice and is highly expressed in NSCLC patients. **A-D**, RASON knockout suppressed KRAS-driven lung tumorigenesis in *LSL-Kras*^*G12D*^; *LSL-Trp53*^*R172H/+*^ (KP) mice. **A**, Representative micro-CT images and H&E-stained lung tissue sections from KP and KPR (KP mice with Rason knockout) mice (*n* = 6 for KP, *n* = 4 for KPR, scale bar, 2 mm). **B**, Representative images of lungs from KP and KPR mice. Black circles indicate lung tumor. Scale bar, 1 cm. **C**, Quantification of lung tumor areas in KP and KPR mice. **D**, Kaplan-Meier survival curve analysis of KP and KPR mice (*n* = 12 for KP, *n* = 10 for KPR). **E-K**, RASON expression is elevated in a tissue microarray of 80 human NSCLC samples. **E**, Representative IHC images of RASON staining in NSCLC tumor tissues and adjacent normal tissues. Scale bar, 50 μm. **F**, Dot plot showing quantified H-scores for RASON expression. **G**, Representative IHC images showing RASON expression in NSCLC samples across different tumor stages. Scale bar, 50 μm. **H**, Dot plot showing H-score of RASON expression for KRAS^mut^ NSCLC samples (*n* = 44) stratified by tumor stages. **I**, Dot plot showing H-score of RASON expression for KRAS^mut^ NSCLC samples stratified by combined stages. **J**, Correlation analysis of RASON expression and CD11b levels in KRAS^mut^ NSCLC samples. **K**, Kaplan–Meier survival analysis of KRAS^mut^ NSCLC patients stratified into high and low RASON expression groups based on H-scores (*n* = 20 for the RASON-low group, *n* = 24 for the RASON-high group). Data are shown as mean ± S.E.M. and analyzed by *Student’s t-test* (**C**, **F**, **I**) or *one-way ANOVA* (**H**). * *p* < 0.05; ** *p* < 0.01; *** *p* < 0.001; **** *p* < 0.0001

To investigate the role of RASON in the development of lung cancer, we generated a mouse model in which the start codon (ATG) in the RASON open reading frame (ORF) was substituted with a stop codon (TAA), i.e., *Rason*^*mut/mut*^ mice (Fig. S1A). Using CRISPR-mediated gene editing on *LSL-Kras*^*G12D*^; *LSL-Trp53*^*R172H/+*^ (KP) mice and in vitro fertilization, we established the *LSL-Kras*^*G12D*^; *LSL-Trp53*^*R172H/+*^; *Rason*^*mut/mut*^ (KPR) mouse model (Fig. S1A). To induce autochthonous tumors in the lung, KP and KPR mice were intratracheally infected with adeno-associated virus containing cre (AAV-Cre, 1 × 10^10^ PFU). Lung tumor formation was assessed using micro-CT imaging at 12 weeks post-infection (Fig. S1B). Compared to KP mice, KPR mice exhibited significantly fewer tumor nodules and smaller tumor volumes as determined by micro-CT imaging and H&E staining (Fig. 1A-C and Fig. S2A-B). Additionally, *Rason* knockout (KO) significantly prolonged survival in KPR mice (Fig. 1D), suggesting that RASON is critical for lung tumor formation in the KP model. Immunohistochemistry (IHC) analysis further revealed reduced p-AKT and p-ERK levels, key markers of KRAS downstream signaling, and increased macrophage infiltration in KPR lung tumors compared to KP tumors (Fig. S2C-D).

### RASON is highly expressed in NSCLC

To evaluate whether RASON could serve as a prognosis marker in NSCLC, we performed IHC staining on a tissue microarray containing 80 NSCLC samples. RASON expression was significantly higher in lung cancer tissues compared to adjacent normal tissues (Fig. 1E-F). Further analysis revealed a positive correlation between RASON expression levels and tumor stage (Fig. S2E-F). Pearson’s correlation coefficient analysis demonstrated a strong positive correlation between RASON and p-AKT levels, with a coincident expression pattern observed in patient tissues (Fig. S2G-H). Although survival analysis did not achieve statistical significance, elevated RASON expression was associated with a trend toward poorer overall outcomes in this cohort (Fig. S2I).

To investigate the correlation between RASON expression and KRAS mutations, we used another patient cohort comprising 44 KRAS-mutant and 91 KRAS wild-type NSCLC patients. In KRAS-mutant patients, RASON expression positively correlated with tumor stage (Fig. 1G-I) and negatively correlated with macrophage infiltration in KRAS-mutant patients (Fig. 1J). Survival analysis further revealed that higher RASON expression was linked to shorter survival in KRAS-mutant patients (Fig. 1K), In contrast, RASON expression was not associated with tumor stage, macrophage infiltration, or survival in KRAS wild-type patients (Fig. S2J-N).

Together, these findings confirm that RASON plays a critical role in KRAS-driven lung cancer, driving tumorigenesis and malignant progression.

### RASON promotes oncogenic KRAS^G12C^ signaling and tumor growth in NSCLC cell lines

**Fig. 2:**
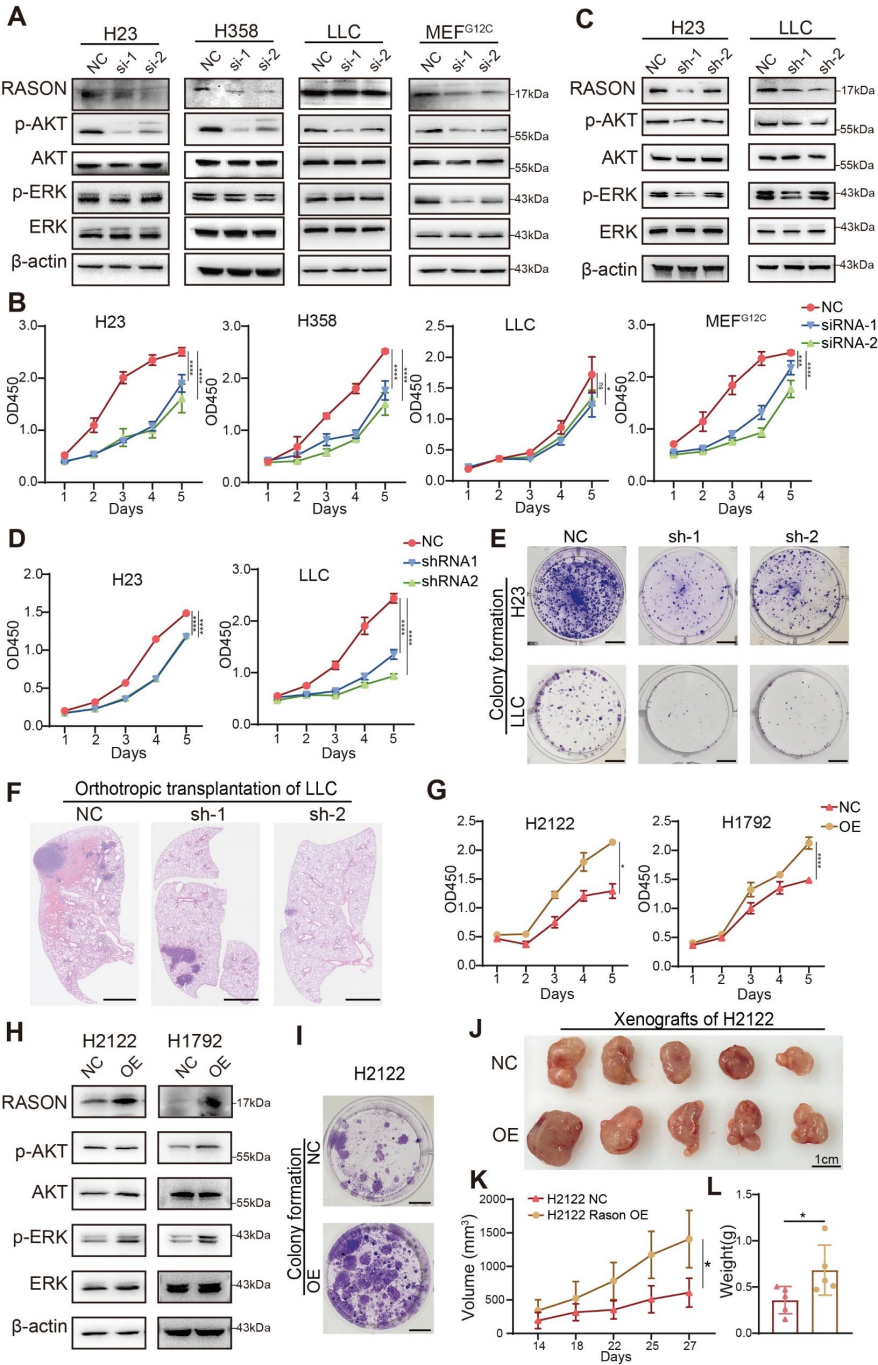
RASON promotes oncogenic KRAS^G12C^ signaling and tumor growth in NSCLC cell lines. **A**, Effects of transient RASON knockdown (KD) by siRNA on KRAS downstream signaling pathway in KRAS^G12C^ lung cancer cell lines and KRAS^G12C^-transformed RAS-less MEFs. **B**, Effects of RASON KD by siRNA on cell proliferation. **C**, Effects of stable RASON knockdown (KD) by shRNA on KRAS downstream signaling pathway in the H23 and LLC lung cancer cell lines. **D**, Effects of RASON KD by shRNA on the proliferation of H23 and LLC cell lines. **E**, Representative images of colony formation with or without RASON KD. Scale bar, 1 cm. **F**, Representative H&E staining images of lung sections derived from LLC lung colonization models with or without RASON KD. Scale bar, 50 μm. **G**, Effects of RASON overexpression (OE) on the proliferation of H2122 and H1792 cell lines. **H**, Effects of RASON OE on KRAS downstream signaling pathways in H2122 and H1792 cell lines. **I**, Representative images of colony formation with or without RASON OE. Scale bar, 1 cm. **J-L**, Effects of RASON OE on in vivo tumor growth of H2122 cells in nude mice. **J**, Representative images of xenografts from H2122 cells with or without RASON OE. Scale bar, 1 cm. **K-L**, Quantitative analysis of tumor weights and volumes in xenograft models (*n* = 8). Data are shown as mean ± S.D. and analyzed by *Student’s t-test* (G, K, L) or *one-way ANOVA* (B, D). * *p* < 0.05; ** *p* < 0.01; *** *p* < 0.001; **** *p* < 0.0001

Given that KRAS^G12C^ mutation is the most prevalent KRAS mutation in lung cancer, we investigated whether RASON regulates KRAS^G12C^ activity and its oncogenic functions in NSCLC. We first assessed RASON expression in four human KRAS^G12C^ mutant NSCLC cell lines (H23, H358, H1792, and H2122) (Fig. S3A). Among these cell lines, RASON expression was highest in H358 and H23 cells but relatively low in H1792 and H2122 cells. Transient knockdown (KD) of RASON using siRNA in human cells (H358, H23), KRAS^G12C^ mutant mouse lung cancer cells (LLC) and KRAS^G12C^-transformed Ras-less mouse embryonic fibroblast cells (MEF^G12C^) resulted in significant downregulation of KRAS downstream signaling pathways, including the MAPK (RAF-MEK-ERK) and PI3K-AKT pathways (Fig. 2A). Cell proliferation assays using CCK8 showed that RASON silencing suppressed cell proliferation across all four cell lines (Fig. 2B). To evaluate the in vivo effects of RASON silencing, we used shRNA-mediated knockdown in H23 and LLC cells, followed by mouse xenograft and lung colonization assays, respectively. Immunoblotting confirmed that RASON knockdown reduced MAPK and PI3K-AKT signaling (Fig. 2C), while functional assays demonstrated a significant reduction in 2D colony formation in vitro and tumor growth in vivo (Fig. 2D-F and Fig. S3B). Conversely, overexpression of RASON-ORF in RASON low-expressing H1792 and H2122 cells enhanced MAPK and PI3K-AKT signaling, promoted cell proliferation and colony formation in vitro, and accelerated tumor growth in vivo (Fig. 2G-L and Fig. S3C).

These findings demonstrate that RASON is a critical regulator of KRAS^G12C^ effector signaling in NSCLC. Silencing RASON significantly inhibits tumor growth, highlighting its pivotal role in KRAS^G12C^ mutant NSCLC progression both in vivo and in vitro.

### RASON directly binds to KRAS^G12C^ and stabilizes KRAS in the GTP-bound hyperactive state

**Fig. 3:**
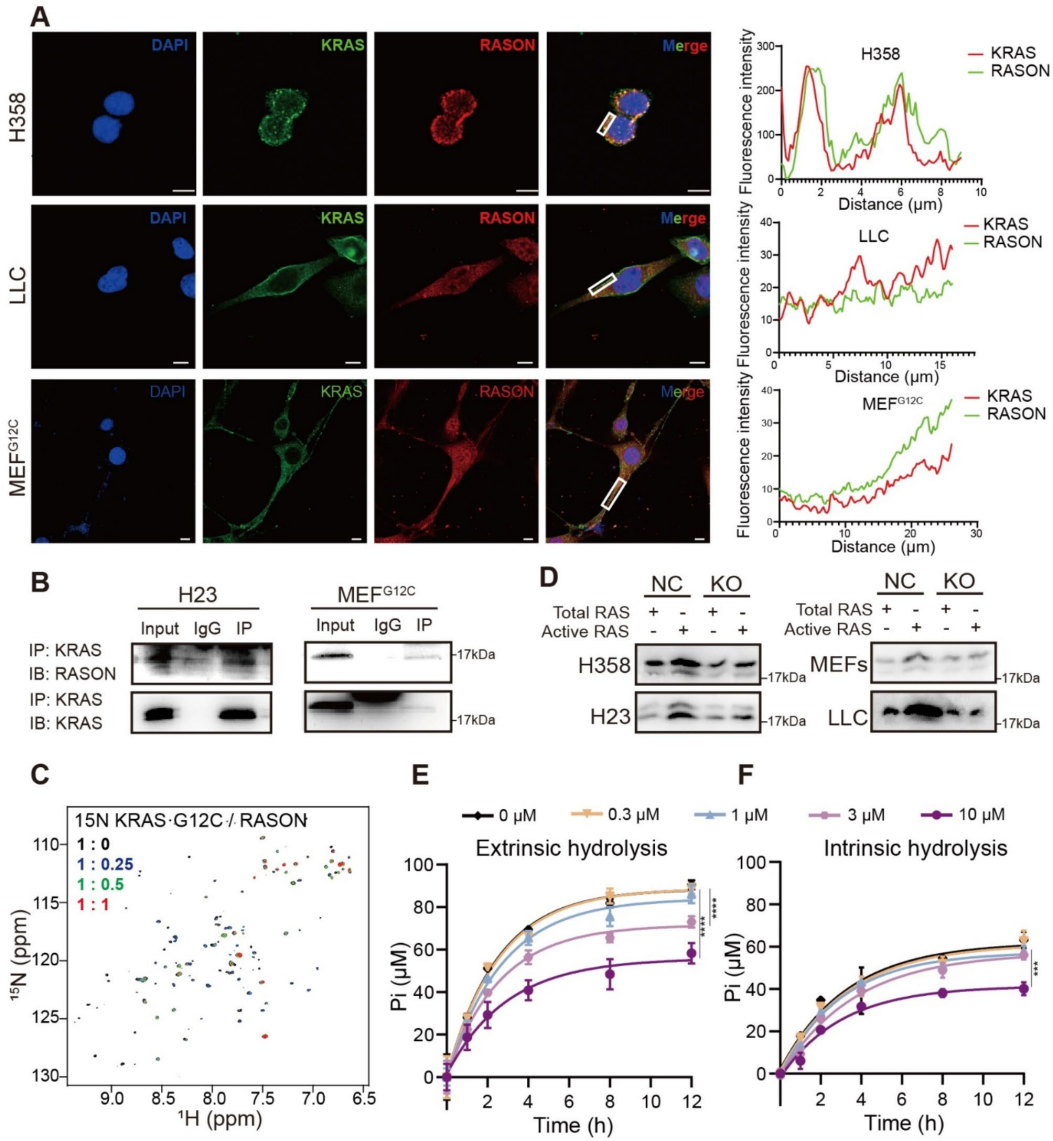
RASON binds to KRAS^G12C^ and stabilizes it in the GTP-bound hyperactive state. **A**, Immunofluorescence staining showing the co-localization of RASON and KRAS in KRAS^G12C^ lung cancer cell lines H358 and LLC, as well as in KRAS^G12C^-transformed RAS-less MEF (MEF^G12C^) cells. Quantification of fluorescence intensity is shown on the right. Scale bar, 10 μm. **B**, Co-immunoprecipitation assay showing the interaction between RASON and KRAS^G12C^ in H23 and MEF^G12C^ cell lines. **C**, 2D ^1^H-^15^N  HSQC spectra of ^15^N-labeled human KRAS^G12C^ in the absence and presence of RASON at the indicated molar ratios. **D**, GST-Raf1-RBD pull-down assay illustrating reduced levels of active GTP-bound KRAS (active RAS) in RASON-KO KRAS^G12C^ lung cancer cells. **E**, NF1-stimulated extrinsic KRAS^G12C^ GTPase activity in the presence of RASON measured by GTPase hydrolysis assay (RASON concentrations indicated in the legend). **F**, Intrinsic KRAS^G12C^ GTPase activity in the presence of RASON measured by GTPase hydrolysis assay (RASON concentrations indicated in the legend). Data shown are means ± S.D. and analyzed by *two-way ANOVA* (E, F). *** *p* < 0.001; **** *p* < 0.0001

Our previous studies have shown that RASON maintains KRAS^G12D^ and KRAS^G12V^ in a hyperactive state by directly binding to these KRAS mutants and stabilizing them in the GTP-bound active forms [31]. To investigate whether RASON similarly interacts with KRAS^G12C^, we performed immunofluorescence staining in KRAS^G12C^ mutant MEF^G12C^ cells and lung cancer cell lines. The results revealed co-localization of RASON with KRAS^G12C^ (Fig. 3A). The direct interaction between RASON and KRAS^G12C^ was further confirmed through co-immunoprecipitation (IP) assays in H23, H358, MEF^G12C^, and LLC cells (Fig. 3B and Fig. S4A). To validate the binding more precisely, we performed Nuclear Magnetic Resonance (NMR) titration experiments. ^15^N-labeled KRAS^G12C^ protein was mixed with unlabeled human RASON protein at molar ratios from 1:0 to 1:1. A dose-dependent decrease in peak intensities was observed in the 2D ^1^H-^15^N heteronuclear single quantum coherence (HSQC) spectra of KRAS^G12C^ in the presence of RASON (Fig. 3C), indicating a strong interaction between the two proteins. To investigate the functional impact of RASON binding, we generated RASON knockout mono-clones in KRAS^G12C^ mutant cell lines H23, H358, LLC, and MEF^G12C^ using CRISPR/Cas9. The levels of active KRAS-GTP were significantly downregulated in RASON KO clones compared to their respective controls (Fig. 3D).

As we previously reported, RASON directly binds and stabilize KRAS-G12D and G12V in their active, GTP-bound state by competing with NF1 [31]. We thus conducted biochemical experiments to demonstrate whether RASON similarly regulates KRAS-G12C. GTPase hydrolysis and nucleotide exchange assays revealed that RASON could inhibit both intrinsic and extrinsic GTPase activity of KRAS^G12C^, while had no effect on its nucleotide exchange (Fig. 3E-F and Fig. S4B-C).

To further elucidate the molecular mechanisms underlying the protumor function of RASON, we performed RNA sequencing (RNA-seq) on H358 parental (NC) and RASON-KO cells (Fig. S5A). KEGG pathway analysis revealed significant changes in KRAS downstream signaling pathways, including the PI3K-AKT pathways (Fig. S5B-C). Gene Set Enrichment Analysis (GSEA) indicated that KRAS dependency genes were downregulated in RASON-KO cells (Fig. S5D-H). Notably, the human gene set “SWEET KRAS TARGETS UP”, comprising genes upregulated upon KRAS knockdown, was enriched in RASON-KO H358 cells compared to controls, suggesting that targeting RASON mimics the effect of KRAS inhibition (Fig. S5I-J). Together, these results demonstrate that RASON directly binds to KRAS^G12C^, stabilizes it in the hyperactive GTP-bound state, and modulates its downstream signaling pathways, contributing to its protumor activity.

### RASON knockout inhibits lung cancer progression and restores macrophage infiltration in vivo

**Fig. 4:**
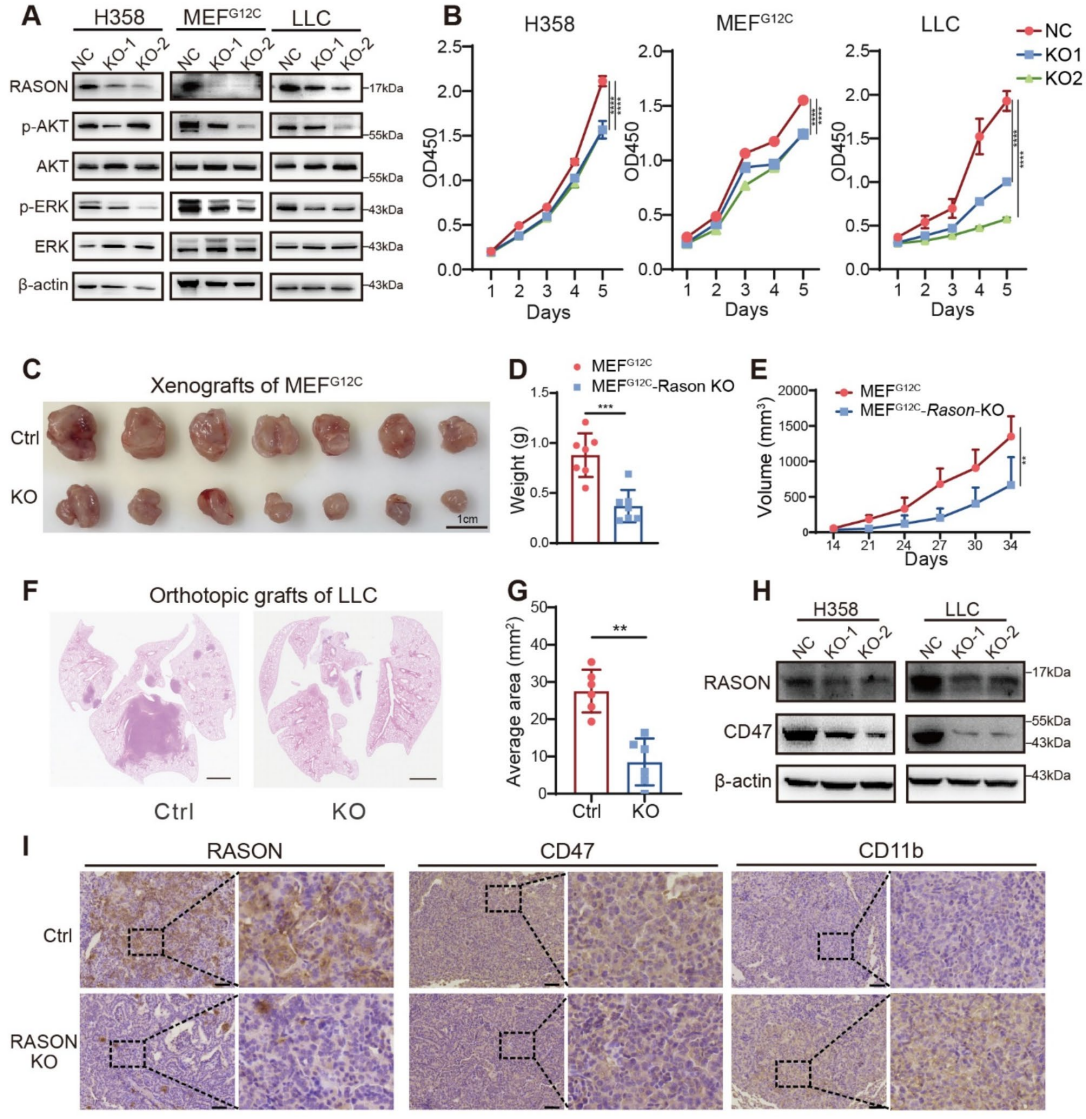
RASON knockout inhibits lung cancer progression in vivo and restores macrophage infiltration. **A**, Effects of RASON KO on KRAS signaling pathways in MEF^G12C^, H358, and LLC cell lines. **B**, Effects of RASON KO on in vitro proliferation of MEF^G12C^, H358, and LLC cell lines. **C-E**, Effects of RASON KO on H358-derived xenograft tumor growth in vivo. (**C**) Representative images of H358 xenograft tumors in nude mice. Scale bar, 1 cm. (**D**, **E**) Quantitative analysis of tumor weights and volumes (*n* = 7 per group). **F-G**, Effects of RASON KO on LLC lung colonization formation in vivo. **F**, Representative H&E staining images of lung sections from LLC lung colonization models in C57B6/J mice. Scale bar, 2 mm. **G**, Quantitative analysis of tumor area (*n* = 6). **H**, Effects of RASON KO on CD47 expression in H358 and LLC cell lines. **I**, IHC staining showing CD11b and CD47 expression levels in LLC-derived lung tumor tissues. Scale bar, 50 μm. Data are shown as mean ± S.D. and analyzed by *Student’s t-test* (**D**, **E**, **G**) or *one-way ANOVA* (**B**, **E**). * *p* < 0.05; ** *p* < 0.01; *** *p* < 0.001; **** *p* < 0.0001

Next, we investigated whether RASON knockout could phenocopy the effects of RASON silencing by siRNA or shRNA in lung cancer cell lines. To this end, we used the human lung cancer cell line H358, the murine lung cancer cell line LLC, and MEF^G12C^ cells. RASON-KO inhibited KRAS downstream signaling (Fig. 4A), reduced in vitro proliferation (Fig. 4B) and suppressed in vivo tumor growth (Fig. 4C-G and Fig. S6A-D) in all three models. Notably, H358 RASON-KO cells completely lost their ability to form xenograft tumors in nude mice (Fig. S6A), despite only partial inhibition of cell proliferation in vitro (Fig. 4B), suggesting a possible involvement of the tumor immune microenvironment in vivo.

To explore this possibility, we performed IHC staining using antibody against CD11b, a marker of macrophage, to evaluate macrophage infiltration in LLC-derived lung tumors in C57B6/J mice. Compared to the control group, a significant increase in macrophage infiltration was observed in LLC RASON-KO tumors (Fig. 4I). Previous work from our lab demonstrated that KRAS-mediated upregulation of CD47 expression drives innate immune evasion in NSCLC [34]. Therefore, we hypothesized that RASON KO might downregulate CD47 expression via modulation of KRAS signaling. Supporting this hypothesis, immunoblotting of tumor cell lysates and IHC analysis of in vivo LLC graft tissues revealed reduced CD47 expression and increased staining of CD11b in RASON KO tumors (Fig. 4H-I), which was consistent with the increased infiltration of macrophages in RASON^low^ human samples (Fig. 1J). This finding was further validated in genetically engineered lung cancer models (KP and KPR mice) (Fig. S6E-F).

Taken together, these results indicate that RASON upregulates KRAS signaling and suppresses macrophage infiltration via upregulation of CD47, thereby promoting lung cancer progression and facilitating innate immune evasion.

### RASON promotes tumor evasion from macrophage phagocytosis

**Fig. 5:**
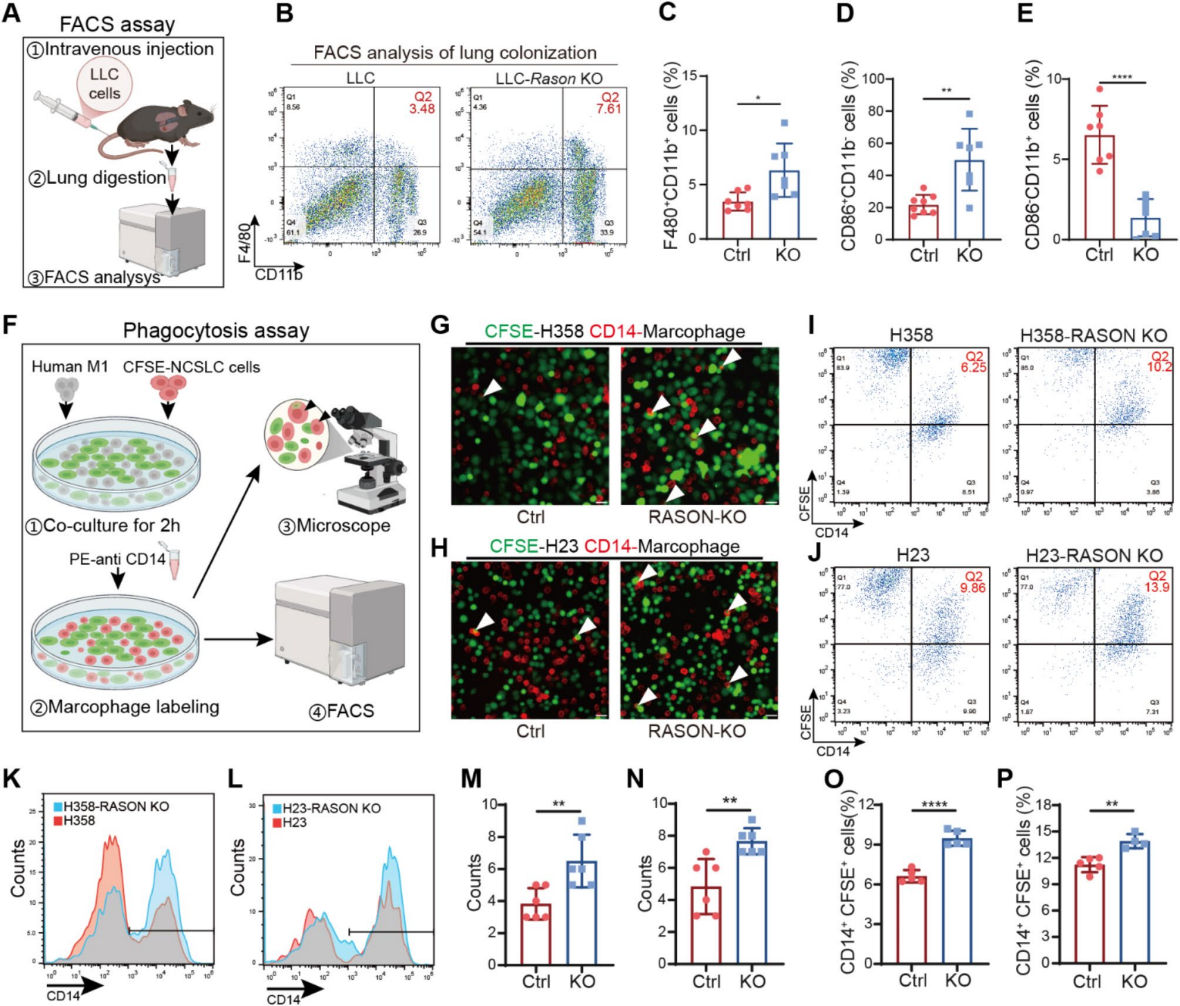
RASON knockout promotes macrophage phagocytosis of lung cancer cell lines. **A-E**, Effects of RASON KO on macrophage infiltration in LLC lung colonization tumors. **A**, Schematic representation of the FACS assay. Cells isolated from LLC lung colonization tumors were labelled antibodies against with antibodies against CD45, F4/80, CD11b, CD86, and CD206, and sorted using fluorescence-activated cell sorting (FACS). The percentage of activated macrophage cells (**A-C**), as well as M1 and M2 macrophages (**E-F**), was analyzed. CD45 + F480 + CD11b + cells represent activated macrophage cells, CD45 + F480 + CD11b + CD86 + CD11b- cells represent M1 macrophages, and CD45 + F480 + CD11b + CD86-CD11b + represent M2 macrophages. **F-P**, Effects of RASON KO on macrophage-mediated in vitro phagocytosis. (**F**) Schematic representation of the macrophage phagocytosis assay. Co-localization of macrophages with tumor cells in immunofluorescence staining and double-positive fluorescence signals in FACS analysis were used to indicate phagocytosis activity. (**G-H**) Representative immunofluorescence images of macrophage phagocytosis of tumor cells with or without RASON KO. (**I-L**) Representative percentage analysis of CFSE + CD14 + cells among the CFSE + cells (**I**, **J**) and mean fluorescence intensity of CD14 in CFSE + cells (**K**, **L**); (**M-N**) Quantitative analysis of G and H. (**O-P)**, Quantitative analysis of I and J. Data are shown as mean ± S.D. and analyzed by *Student’s t-test* (C-E, M-P). * *p* < 0.05; ** *p* < 0.01; **** *p* < 0.0001

To further investigate the effect of RASON knockout on tumor immune evasion, we performed flow cytometry to analyze the infiltration of macrophages and T cells in LLC lung colonization models (Fig. 5A). Immune cell subsets were identified using the following cell surface markers: (i) classically activated macrophages (M1) as CD45^high^CD11b^high^F4/80^high^CD86^high^CD206^low^ cells; (ii) alternatively activated macrophages (M2) as CD45^high^CD11b^high^F4/80^high^CD86^low^CD206^high^ cells; (iii) cytotoxic T cells as CD45^high^CD11b^high^CD3^high^CD4^low^CD8^high^ cells; (iv) T helper cells as CD45^high^CD11b^high^CD3^high^CD4^high^CD8^low^cells. The gating strategy is outlined in Fig. S7A. The results revealed a significant increase in the infiltration of activated macrophages (Fig. 5B-C) and M1 macrophages (Fig. 5D) in RASON KO lung colonization tumors, along with a decrease in M2 macrophages (Fig. 5E). However, no significant changes were observed in the infiltration of T cells, including CD8^+^ cytotoxic T cells and CD4^+^ T helper cells (Fig. S7B-F). These results align with the absence of tumor formation in nude mice injected with H358 cells (Fig. S6A), which lack functional T cells due to the absence of a thymus.

Next, we evaluated the effect of RASON expression on macrophage-mediated tumor phagocytosis. CFSE-labeled lung cancer cells were co-cultured with CD14-labeled human peripheral blood monocyte-derived M1 macrophages. The number of phagocytosed cancer cells was quantified using FACS and fluorescence microscopy (Fig. 5F). In RASON^high^ cell lines (H358 and H23), RASON KO significantly increased macrophage-mediated phagocytosis (Fig. 5G-P). Conversely, RASON OE in the RASON^low^ cell line H1792 reduced phagocytosis (Fig. S7G-I).

Together, these results demonstrate that RASON activates CD47 expression in cancer cells and inhibits macrophage-mediated phagocytosis, thereby enabling innate immune evasion and driving aggressive tumor progression.

### RASON is a potential therapeutic target for KRAS^G12C^ mutant NSCLC

**Fig. 6:**
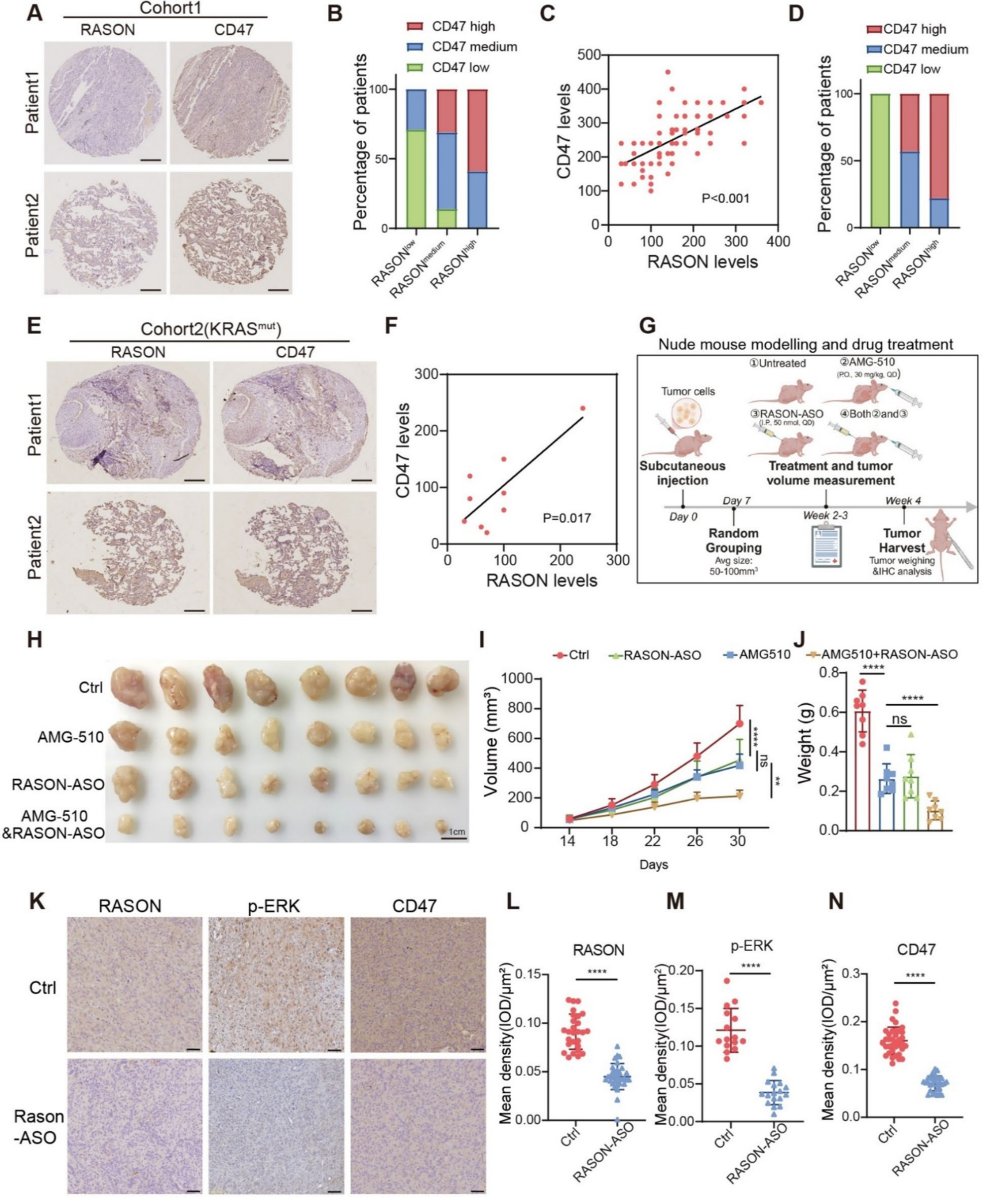
RASON is a potential therapeutic target for KRAS^G12C^ non-small cell lung cancer. **A**, Representative IHC staining images of RASON and CD47 expression in NSCLC patient cohort 1 (*n* = 80). Scale bar, 50 μm. **B**, Bar graph showing CD47 expression levels in NSCLC patients stratified by RASON expression in cohort 1 (RASON^low^, *n* = 24; RASON^medium^, *n* = 29; RASON^high^, *n* = 27). **C**, Pearson’s correlation coefficient analysis of the correlation between the expression levels of CD47 and RASON in NSCLC patient cohort 1. **D**, Bar graph showing CD47 expression levels in NSCLC patient cohort 1 with high p-AKT expression, stratified by RASON expression (RASON^low^, *n* = 1; RASON^medium^, *n* = 7; RASON^high^, *n* = 18). **E**, Representative IHC staining images of RASON and CD47 expression in KRAS mutant NSCLC patient cohort 2 (*n* = 9). Scale bar, 50 μm. **F**, Pearson’s correlation coefficient analysis of the correlation between the expression levels of CD47 and RASON in NSCLC patient cohort 2 (*n* = 9). **G**, Schematic diagram of nude mouse modelling and drug treatment. H358 and H23 xenograft tumors were treated with either AMG-510, RASON-ASO, or both. **H-J**, Synergetic effect of RASON antisense oligonucleotides (ASO) and AMG-510 treatment in H358 xenograft tumors. (**H**) Representative xenograft images. Scale bar, 1 cm. (**I**) Quantitative analysis of tumor volumes. (**J**) Quantitative analysis of tumor weights. **K-N**, IHC staining (**K**) and quantitative analysis of RASON (**L**), KRAS downstream signal p-ERK (M) and the immune marker CD47 (**N**). Scale bar, 50 μm. Data are shown as mean ± S.D. and analyzed by *Student’s t-test* (**J**, **L**, **M**, **N**) or *one-way ANOVA* (**I**). * *p* < 0.05; ** *p* < 0.01; *** *p* < 0.001; **** *p* < 0.0001

To validate the clinical relevance of our findings, we assessed the correlation between RASON and CD47 expression in two independent lung adenocarcinoma cohorts. In the first cohort, a tissue microarray containing 80 NSCLC samples was analyzed using IHC staining. Patient samples were categorized into three groups based on high, medium, and low levels of RASON expression. As expected, CD47 expression was highest in the group with high RASON levels (Fig. 6A-B). Pearson’s correlation coefficient analysis confirmed a positive correlation between RASON and CD47 expression (Fig. 6C). To evaluate this relationship in the context of KRAS hyperactivation (KRAS mutation status is not available for this cohort), we used p-AKT levels, a downstream effector of PI3K signaling, as a surrogate marker for KRAS activity. In the high p-AKT group, a strong correlation between RASON and CD47 expression was also observed (Fig. 6D). In the second cohort, we performed IHC analysis of CD47 and RASON expression on an in-house-generated tissue microarray containing paired tumor and adjacent normal tissue samples from nine patients with KRAS-mutant lung adenocarcinoma. Consistent with the first cohort, a strong correlation between RASON and CD47 expression was observed in these samples (Fig. 6E-F).

Given the clinical challenges posed by the resistance to KRAS^G12C^ inhibitors, we explored whether RASON inhibition could synergize with KRAS^G12C^-targeting therapy. Therapeutic experiments were conducted using AMG-510, an FDA-approved KRAS^G12C^ inhibitor, and RASON antisense oligonucleotides (RASON-ASO) in vivo (Fig. 6G). Xenograft models were established by subcutaneously injecting human lung cancer cells (H358 and H23) into BALB/c Nude mice. Upon tumor formation, mice were randomly assigned to one of four treatment groups: control, AMG-510 monotherapy (p.o., 30 mg/kg, QD), RASON-ASO monotherapy (i.p., 50 nmol, QD), and the combination therapy with AMG-510 and RASON-ASO.

Compared to the control group, RASON-ASO treatment showed a similar inhibition effect on tumor growth as AMG-510 (Fig. 6H-J and Fig. S8A-C), and the combination of AMG-510 and RASON-ASO resulted in a significantly enhanced suppression effect (Fig. 6H-J and Fig. S8A-C), indicating that RASON inhibition synergizes with AMG-510 for clinical treatment. IHC staining results further revealed that RASON-ASO treatment not only inhibited cell proliferation (evidenced by decreased ki-67 staining) but also increased macrophage infiltration (CD11b+) and down-regulated CD47 expression (Fig. 6K-N and Fig. S8D-G).

Taken together, these findings demonstrate that RASON is a promising therapeutic target and a potential synergetic factor for KRAS^G12C^ inhibitors in the treatment of NSCLC.

## Discussion

Lung cancer is the most frequently diagnosed malignancy worldwide, with non-small cell lung cancer (NSCLC) accounting for approximately 85% of cases. Alarmingly, about 75% of patients are diagnosed at advanced stages, resulting in a dismal five-year survival rate [4]. KRAS mutations are detected in approximately 30% of NSCLC patients, with KRAS^G12C^ being the most prevalent variant [15]. Targeting KRAS has long been considered one of the most significant yet elusive challenges in oncology. Recent advances have led to the approval of direct inhibitors for KRAS^G12C^, such as AMG-510, marking a breakthrough in the field. However, the clinical efficacy of these inhibitors is constrained by resistance mechanisms, including secondary KRAS mutations, reactivation of upstream or downstream pathways, immune evasion, and tissue-specific mechanisms of resistance [27, 35], underscoring the urgent need to investigate deeper into the molecular mechanisms regulating KRAS^G12C^oncogenic signaling.

Our previous study provides compelling evidence that RASON, a novel protein we discovered in pancreatic cancer, functions as a stabilizer of KRAS activity [31]. Here, we extend these findings to NSCLC, demonstrating that RASON binds and stabilizes KRAS^G12C^, thereby upregulating oncogenic downstream signaling pathways. Knockdown of RASON in NSCLC cells significantly reduces tumor growth in vitro and in vivo, underscoring its role as a positive regulator of KRAS^G12C^ signaling. Furthermore, RASON inhibition enhances the therapeutic efficacy of AMG-510, a KRAS^G12C^ inhibitor, suggesting that RASON targeting could exert anti-tumor effects alone or synergize with existing KRAS-directed therapies to improve treatment outcomes.

**Fig. 7:**
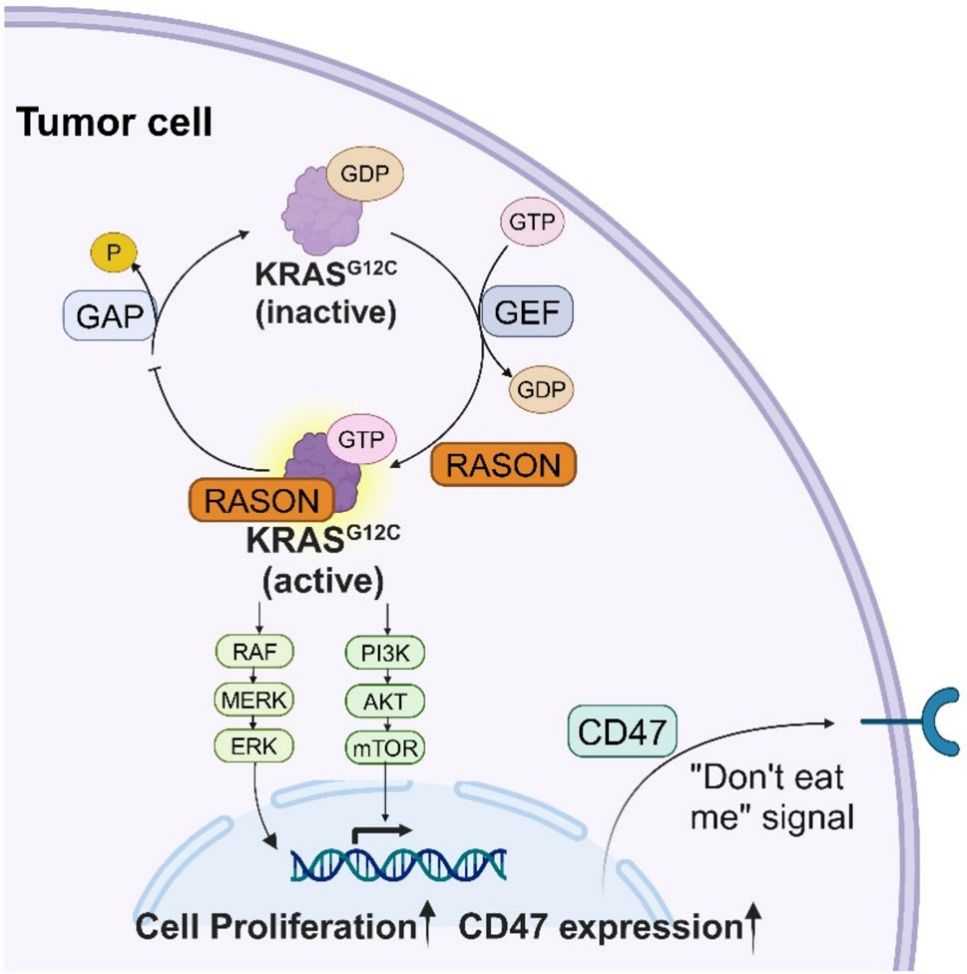
Schematic representation of the pro-tumor mechanisms of RASON in KRAS^G12C^ driven NSCLC. RASON upregulates KRAS^G12C^ downstream signaling to promote cell proliferation and activates CD47 expression to inhibit macrophage phagocytosis (Created with Biorender.com)

In addition to its role in tumor proliferation, we identified RASON as a novel modulator of tumor immune evasion (Fig. 7). Our data revealed that RASON knockdown downregulates CD47 expression, a key “don’t-eat-me” signal that inhibits macrophage-mediated phagocytosis of lung cancer cells [36–38]. Consequently, RASON inhibition enhances macrophage infiltration and immune activation in the tumor microenvironment. However, we did not observe significant changes in T cell infiltration, including CD4 + and CD8 + T cells, indicating that RASON’s immunoregulatory effects may be specific to macrophage-mediated innate immunity. This cell type-specificity warrants further investigation.

Our study advances the understanding of KRAS^G12C^ regulation by demonstrating that RASON is neither a guanine nucleotide exchange factor (GEF) nor a GTPase-activating protein (GAP), but rather a direct binding partner that stabilizes KRAS^G12C^ in its hyperactive GTP-bound state and inhibits its GTPase activity. While classical theories of KRAS regulation focus on the GDP/GTP cycling regulated by GEFs and GAPs, our findings expand the theoretical framework by introducing RASON as a novel positive regulator of KRAS^G12C^. This builds on our earlier work showing RASON’s role in stabilizing KRAS^G12D^ and KRAS^G12V^ in pancreatic cancer and further suggests that RASON may be a universal regulator of mutant KRAS isoforms across cancer types.

Despite these promising findings, several limitations should be acknowledged. First, while our data suggest a direct interaction between RASON and KRAS^G12C^ and its effect on KRAS^G12C^ biochemical properties, the precise molecular mechanism remains to be elucidated. Structural studies are needed to define the binding interface. Second, the tissue-specific immune effects of RASON, particularly its lack of impact on T cell infiltration, highlight the complexity of tumor-immune interactions and call for further studies in different cancer models. Third, the safety and efficacy of RASON-targeting strategies, including antisense oligonucleotides (ASOs), require validation in preclinical and clinical settings. Nevertheless, our study lays the groundwork for exploring RASON as a therapeutic target for KRAS^G12C^-mutant cancers. Combination strategies involving RASON inhibition and KRAS^G12C^ inhibitors like AMG-510 could be particularly promising in enhancing treatment efficacy. Future research should focus on developing RASON-targeting approaches, investigating its role in other KRAS mutations and cancer types, and exploring its potential to enhance responses to immunotherapies.

## Conclusion

Our findings establish RASON as a critical regulator of KRAS^G12C^-driven tumor progression and immune evasion in NSCLC. RASON is a promising therapeutic target for KRAS^G12C^ mutant non-small cell lung cancer either as a monotherapy or in combination with KRAS inhibitors.

## Electronic supplementary material

Below is the link to the electronic supplementary material.

Supplementary Material 1

Supplementary Material 2

## Data Availability

RNA-seq data from this study were deposited in the NCBI Sequence Read Archive (SRA, PRJNA1199328) and will be publicly available as of the date of publication.

## Abbreviations

AAV: Adeno-associated virus
ASO: Antisense oligonucleotides
CFSE: Carboxyfluorescein diacetate succinimidyl ester
DAPI: 4’, 6-diamidino-2-phenylindole
FBS: Fetal bovine serum
GAP: GTPase-activating protein
GEF: Guanine nucleotide exchange factor
GM-CSF: Granulocyte-macrophage colony-stimulating factor
GSEA: Gene Set Enrichment Analysis
H&E: Hematoxylin and eosin
HSQC: Heteronuclear single quantum coherence
IHC: Immunohistochemistry
IP: Immunoprecipitation
KD: Knockdown
KO: Knockout
KP: Kras^LSL-G12D^; Trp53^R172H/+^ mouse
KPR: Kras^LSL-G12D^; Trp53^R172H/+^; Rason^mut/mut^ mouse
LUAD: Lung adenocarcinoma
LUSC: Lung squamous cell carcinoma
MAPK: Mitogen-activated protein kinase
MEF: Mouse embryonic fibroblast cells
micro-CT: Micro-computed tomography
NMR: Nuclear Magnetic Resonance
NSCLC: Non-small cell lung cancer
ORF: Open reading frame
PBMC: Peripheral blood mononuclear cell
PDAC: Pancreatic ductal adenocarcinoma
PI3K: Phosphatidylinositol 3-kinase
PS: Penicillin-streptomycin
RAS: Rat sarcoma gene family
RNAi: RNA interference
RNA-seq: RNA sequencing
SRA: Sequence Read Archive
