# Supplementary material for: RASON promotes KRASG12C-driven tumor progression and immune evasion in non-small cell lung cancer
Source: J Exp Clin Cancer Res. 2025 Mar 25;44:106. doi: 10.1186/s13046-025-03369-9 (PMC11934775; doi:10.1186/s13046-025-03369-9)
Supplement: Supplementary file 1 — Supplementary Material 1 [file 13046_2025_3369_MOESM1_ESM.pdf]

Supplementary Figures for

RASON promotes KRAS^{G12C}-driven tumor progression and immune evasion in non-small cell lung cancer

Jianzhuang Wu, Kexin Xie, Yixuan Zhang, Weiyi Zhang, Rongjie Cheng, Yaliang Zhang, Yugui Xia, Tongyan Liu, Rong Yin, Yudong Qiu, Tao Xu, Rutian Li, Qi Sun, &Chao Yan

Corresponding authors: Chao Yan: yanchao@nju.edu.cn

& Qi Sun: sunqi@njglyy.com

The PDF file include:

Figs. S1 to S8

Other Supplementary Materials for this manuscript include the following:

Supplementary Table S1 to S7.

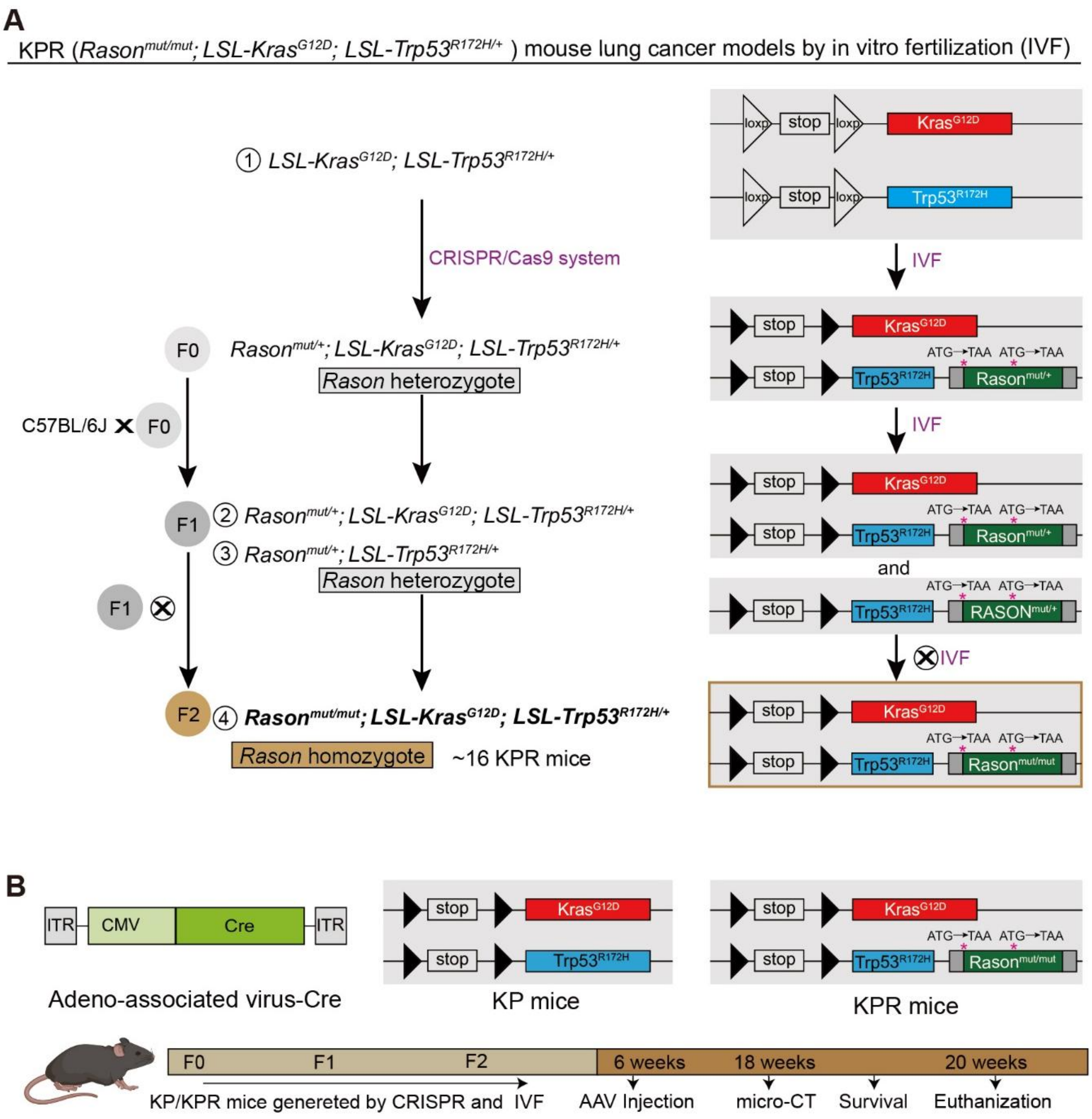

Figure S1, relate to Figure 1

A, Scheme of establishing *Rason*$^{mut/mut}$; *LSL-Kras*^{G12D}; *Trp53*$^{R172H/+}$ mouse model. *Rason*$^{mut/mut}$ mice were crossed with KP mice to generate KPR mice. **B**, Study workflow of lung cancer models generation and experiment timeline. *Kras* and *p53* mutations are activated in lung by intratracheal administration of Adeno-associated virus containing cre to generate lung cancer model in KP and KPR

mice. Mice at the age of 6 weeks are subject to AAV injection to generate lung cancer models, undergo micro-CT testing, survival analysis and finally be euthanatized.

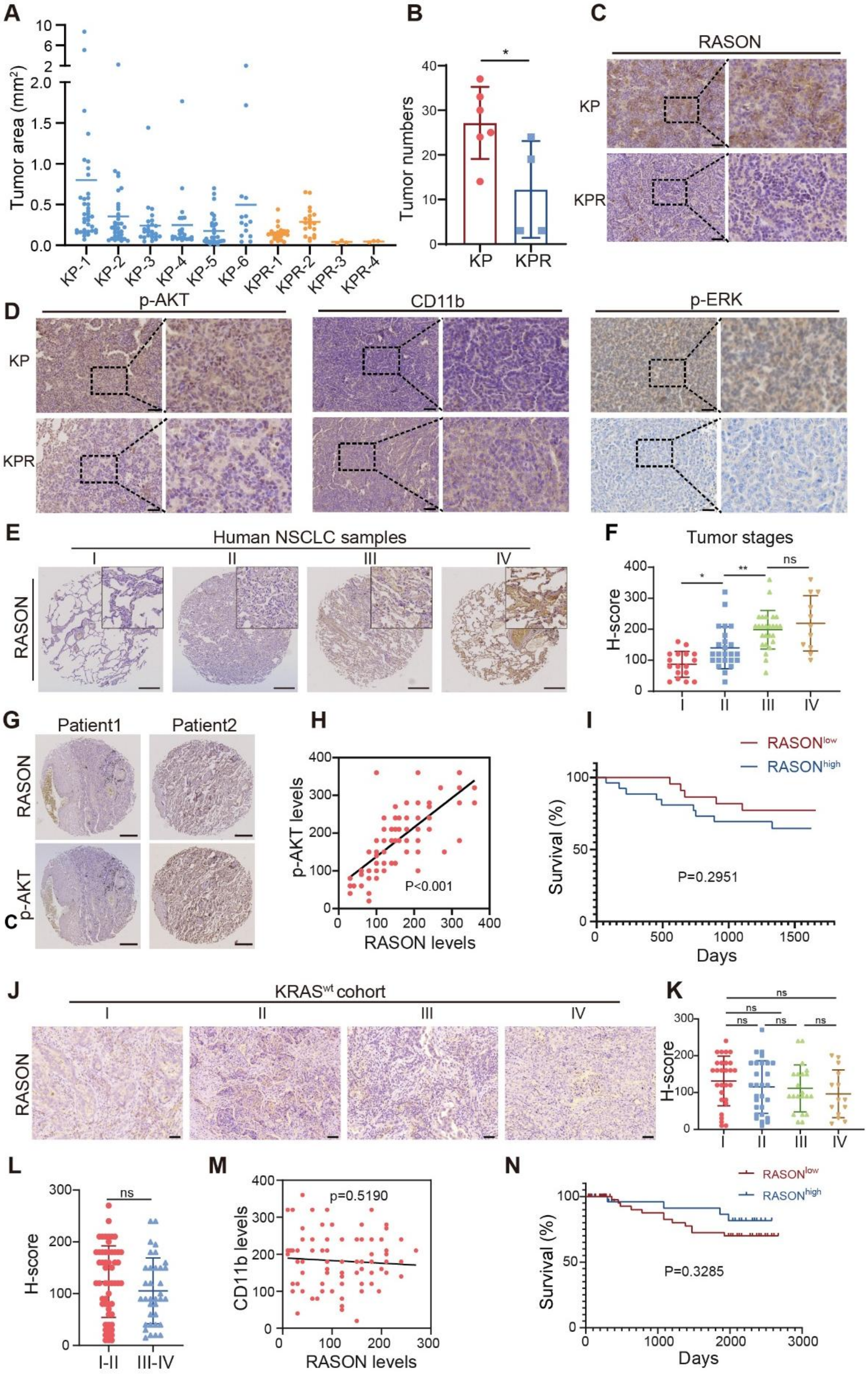

Figure S2, relate to Figure 1

A, Quantification of individual tumor areas of KP and KPR mice. **B**, Quantification of tumor numbers of KP and KPR mice. **C-D**, Representative immunohistochemistry showing p-AKT, p-ERK, and CD11b expression levels in lung tumors of KP and KPR mice. Scale bar, 50 μm. **E**, Representative IHC images showing RASON expression in NSCLC samples across different tumor stages. Scale bar, 50 μm. **F**, Dot plot showing H-score of RASON expression for NSCLC samples at different stages. **G**, Representative IHC images of RASON and p-AKT staining in NSCLC patient tissues. Scale bar, 50 μm. **H**, Correlation analysis of RASON expression and p-AKT levels. **I**, Kaplan–Meier survival analysis of lung adenocarcinoma patients stratified into high and low RASON expression groups based on H-scores (n = 22 for the RASON-low group, n = 27 for the RASON-high group). **J**, Representative IHC images showing RASON expression in $KRAS^{wt}$ NSCLC samples across different tumor stages. Scale bar, 50 μm. **K**, Dot plot showing H-score of RASON expression for $KRAS^{wt}$ NSCLC samples at different stages. **L**, Dot plot showing H-score of RASON expression for $KRAS^{wt}$ NSCLC samples at combined stages. **M**, Correlation analysis of RASON expression and CD11b levels in $KRAS^{wt}$ NSCLC samples. **N**, Kaplan–Meier survival analysis of $KRAS^{wt}$ NSCLC patients stratified into high and low RASON expression groups based on H-scores (n = 53 for the RASON-low group, n = 38 for the RASON-high group). Data are shown as mean ± S.D. and analyzed by *Student's t-test* (B) or *one-way ANOVA* (F). * $p < 0.05$, ** $p < 0.01$.

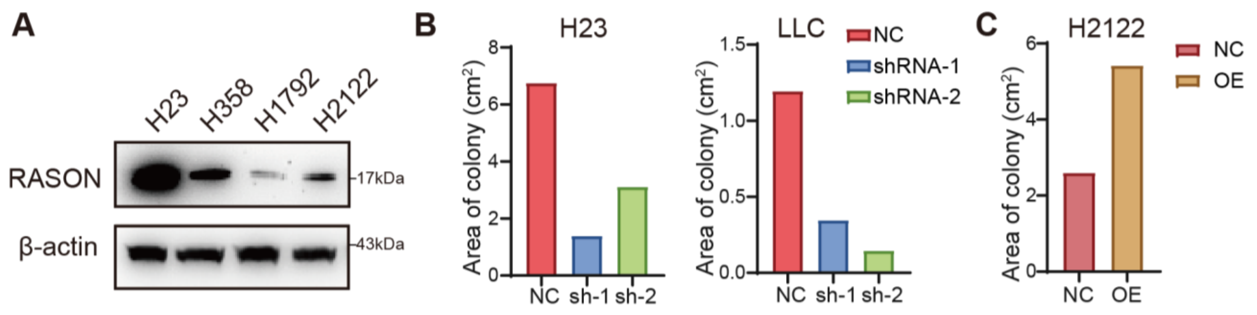

Figure S3, relate to Figure 2

A, Immunoblotting showing the relative RASON expression in human $KRAS^{G12C}$ mutant lung cancer cell lines. **B**, Quantitative analysis of colony areas in H23 and LLC with or without RASON KD. **C**, Quantitative analysis of colony areas in H2122 with or without RASON OE.

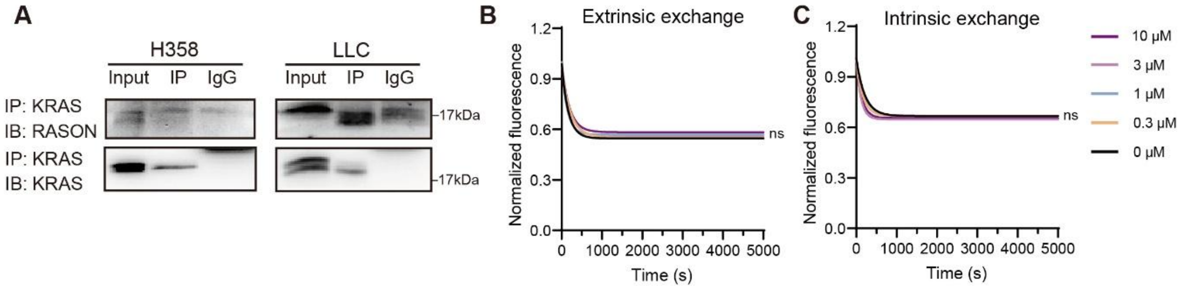

Figure S4, relate to Figure 3

A, Immunoprecipitation (IP) assay showing the mutual binding between RASON and KRAS in LLC.

B-C, Effects of RASON on SOS1-catalysed (B) and intrinsic (C) nucleotide exchange rate of $KRAS^{G12C}$ measured by nucleotide exchange assays.

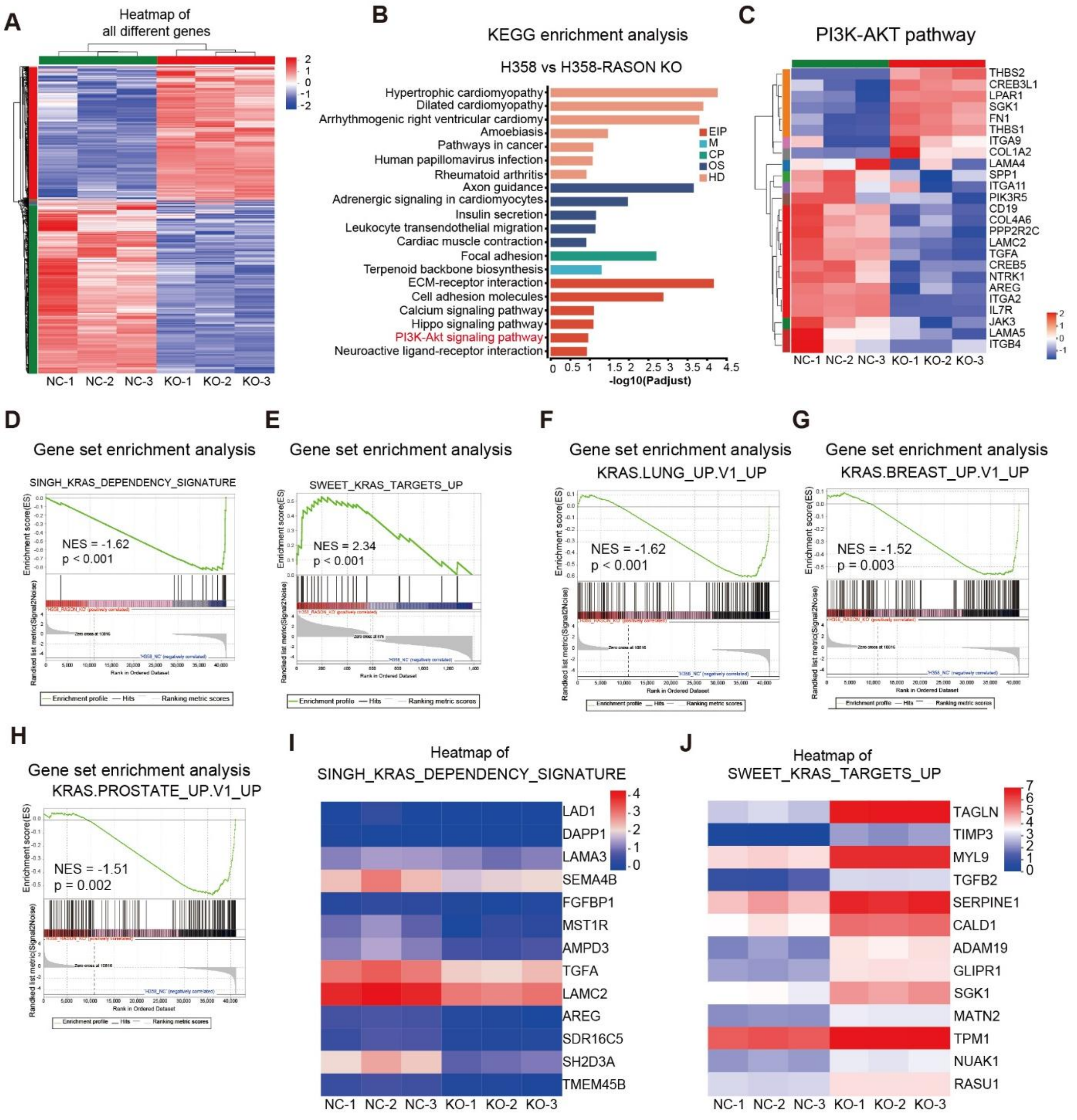

Figure S5, relate to Figure 3

A, Heatmap of Differential Gene Expression analysis of RNA-seq in H358 and H358-RASON KO cells. **B**, KEGG analysis of changes in pathways in H358-RASON KO cells. **C**, Heatmap comparing the expression of PI3K-AKT pathway genes between parental and RASON-KO H358 cells. **D-H**, GSEA plots showing the enrichment of "SWEET_KRAS_TARGETS_UP" and the depletion of the

"SINGH_KRAS_DEPENDENCY_SIGNATURE", "KRAS_LUNG_UP", "KRAS_BREAST_UP", and "KRAS_PROSTATE_UP" signatures in H358 RASON-KO cells. **I**, Expression profiling heatmap of "SINGH_KRAS_DEPENDENCY_SIGNATURE " gene set in H358 and H358-RASON KO cells. **J**, Expression profiling heatmap of "SWEET_KRAS_TARGETS_UP" gene set in H358 and H358-RASON KO cells.

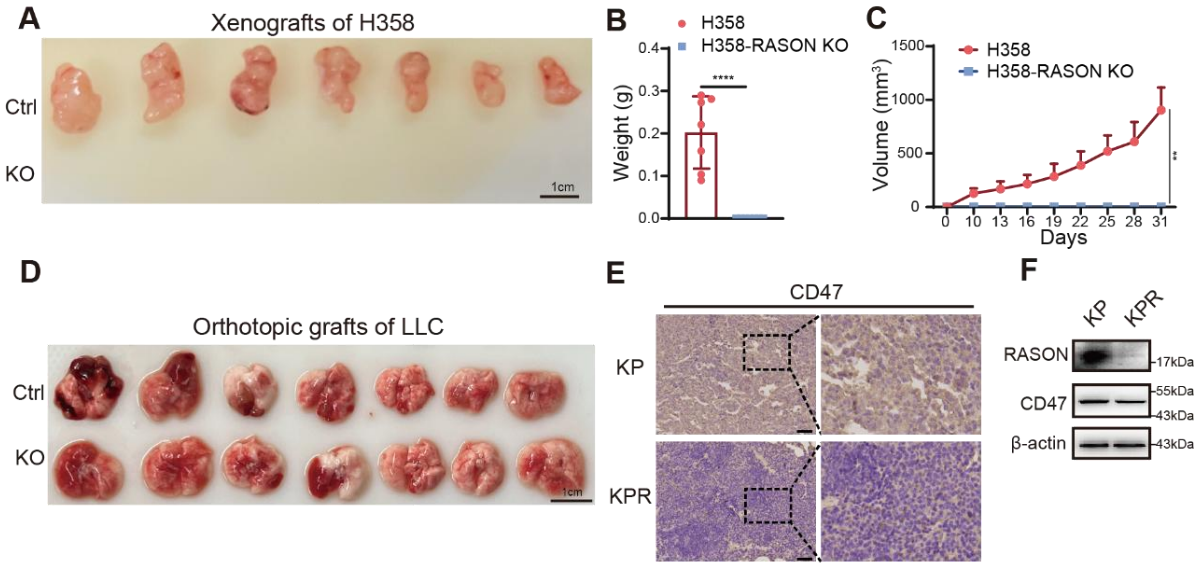

Figure S6, relate to Figure 4

A-C, Effects of RASON KO on subcutaneous MEF^{G12C} tumorigenesis *in vivo*. (A) Representative images of MEF^{G12C}-derived tumors in C57 mice. Scale bar, 1 cm. (B, C) Quantitative analysis of tumor weights and volumes (n = 7 per group). **D**, Representative images of LLC orthotopic grafts with and without RASON KO. Scale bar, 1 cm. **E**, Immunohistochemistry staining showing expression level of CD47 in lung tumors of KP and KPR. Scale bar, 50 μm. **F**, Immunoblotting showing the effect of RASON knockdown on CD47 expression. Data are shown as mean ± S.D. and analyzed by Student's t-test (B, C). ** p<0.01; **** p<0.0001.

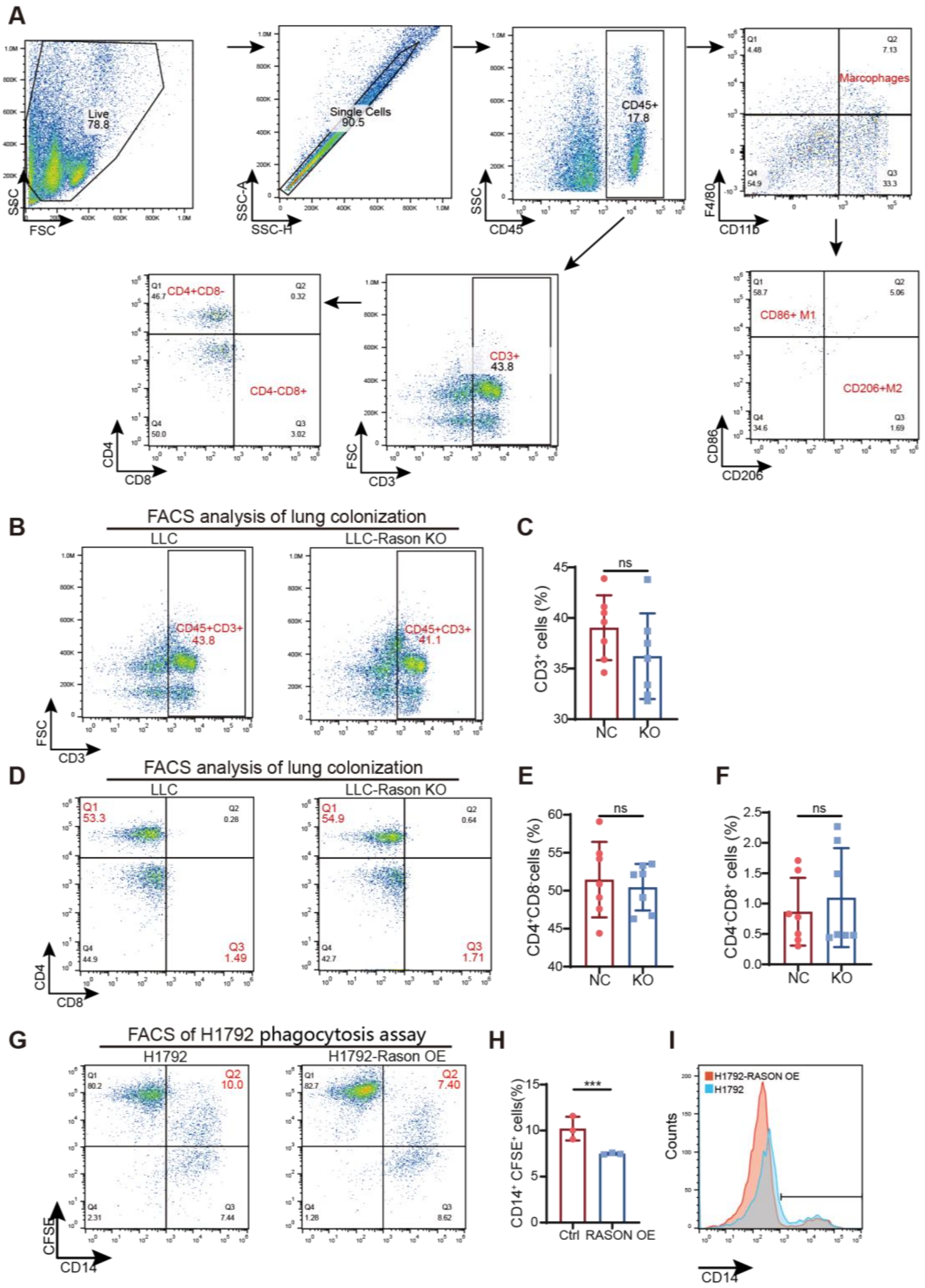

Figure S7, relate to Figure 5

A, Schematic gating strategy of identifying LLC orthotopic graft cells by flow cytometry. **B-C**,

Percentage of CD3+ T cells detected by flow cytometry. Representative FACS images (B) and corresponding quantitative analysis results (C). **D-F**, Percentage of CD4+ and CD8+ T cells detected by flow cytometry. Representative FACS images (D) and corresponding quantitative analysis results of CD4+ T cells (E) and CD8+ T cells (F). **G-I**, Macrophage phagocytosis detected by flow cytometry. Representative FACS images (G) and corresponding quantitative analysis results of phagocytosis occurrence in H2111 and H2122-RASON OE (H, I). Data are shown as mean ± S.D. and analyzed by *Student's t-test* (C, E, F, H). *** $p<0.001$.

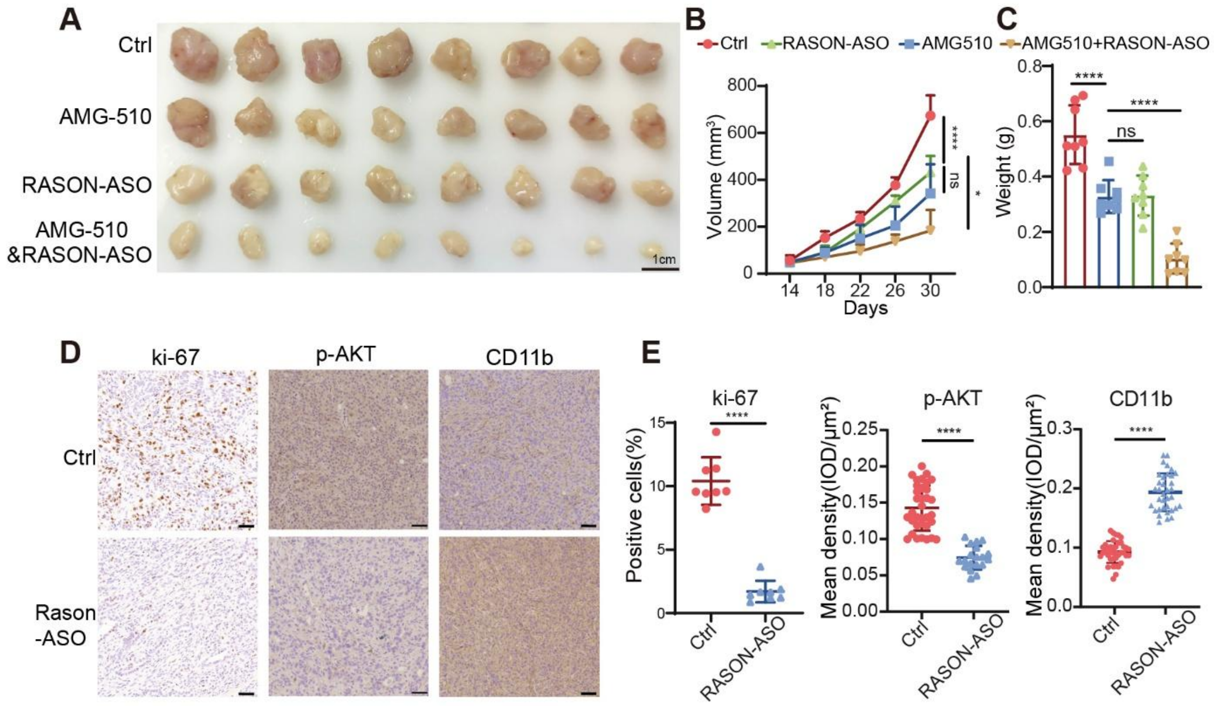

Figure S8, relate to Figure 6

A-C, Synergetic effect of RASON antisense oligonucleotides (ASO) and AMG510 treatment on H23 xenografts. (A) Representative xenograft images. Scale bar, 1 cm. (B) Quantitative analysis of tumor volumes. (B) Quantitative analysis of tumor weights. **D-E**, IHC staining and quantitative analysis of ki-67, p-AKT, and CD11b. Scale bar, 50 µm. Data are shown as mean ± S.D. and analyzed by *one-way ANOVA* (B) or *Student's t-test* (C, E). **** p<0.0001.
